# Observation-based correction of dynamical models using thermostats

**DOI:** 10.1098/rspa.2016.0730

**Published:** 2017-01

**Authors:** Keith W. Myerscough, Jason Frank, Benedict Leimkuhler

**Affiliations:** 1 Department of Computer Science, KU Leuven, 3001 Leuven, Belgium; 2 Mathematical Institute, Utrecht University, PO Box 80010, 3508 TA Utrecht, The Netherlands; 3 School of Mathematics, University of Edinburgh, James Clerk Maxwell Building, Kings Buildings, Edinburgh EH9 3JZ, UK

**Keywords:** sampling, statistical estimation, thermostat, statistical fluid dynamics

## Abstract

Models used in simulation may give accurate short-term trajectories but distort long-term (statistical) properties. In this work, we augment a given approximate model with a control law (a ‘thermostat’) that gently perturbs the dynamical system to target a thermodynamic state consistent with a set of prescribed (possibly evolving) observations. As proof of concept, we provide an example involving a point vortex fluid model on the sphere, for which we show convergence of equilibrium quantities (in the stationary case) and the ability of the thermostat to dynamically track a transient state.

## 1. Introduction

In applications of modern computational science, the underpinning physical laws (and equations of motion) are often well established, yet the detailed behaviour is unpredictable on long time scales due to the presence of deterministic chaos. Examples of this arise in molecular dynamics [1,2], in polymer simulation [3] and in the modelling of turbulent fluids in the atmosphere and ocean [4,5], where long simulations are used to extract statistical information (e.g. the statistics of rare transitions between basins in molecular dynamics, or slow relaxation processes in fluids). In this type of *dynamical sampling*, the common requirement is that simulated paths are sufficiently accurate to calculate measures of dynamical mixing such as 2-point temporal correlation functions [6].

In Hamiltonian systems such as molecular dynamics, it is common to run ensembles of microcanonical (i.e. constant energy) simulations. For such simulations, backward error analysis [7,8] suggests that a long simulation should not be viewed as the approximation of a particular trajectory of the system but rather as an exact solution of a perturbed continuum process described by modified equations. In the case of dynamical sampling of complex systems, the statistics of simulation data are biased because they sample an invariant measure of the modified equations (rather than those of the target system), i.e. the time-discretization error induces an effective statistical bias.

Statistical bias may also arise due to spatial discretization. For example, in the setting of geophysical fluid dynamics, a comparison of discretizations of the quasi-geostrophic equations reveals that the long-time mean potential vorticity field and pointwise fluctuation statistics are heavily dependent on discrete conservation laws such as energy, enstrophy and material conservation of vorticity [9–11]. The discretization bias may be controlled by reducing timestep or by incorporating a Metropolis condition [12,13], possibly increasing the computational cost. The use of a Metropolis condition can also increase the difficulty of computing accurate dynamical properties. The need for computations to be accurate with respect to the dynamical process as well as to the long-term statistics thus poses difficult challenges to the simulator.

In this paper, we consider a method of perturbing the dynamics of the system to correct for statistical bias. Our method is based on the concept of a thermostat, by which we mean a scheme based on a control law designed to facilitate sampling of a target probability density function (pdf). Although originally proposed in molecular dynamics (see, e.g. the Nosé-Hoover method [14,15]), thermostats can be extended to handle a wide variety of systems and have been used in a wide variety of applications. For example, in Dubinkina *et al.* [16], a thermostat was used as a model reduction technique for a vortex model of a fluid (suppressing the detailed interactions of a few strong vortices with a weak vortex field), an approach we draw on in the numerical experiments of this article. In another recent article [17], thermostats have been suggested as a means of sampling incompletely specified systems (with noisy gradients), with applications in machine learning for Bayesian inference in the ‘big data’ context.

In the general modelling scenario, thermostats are significantly hampered by the need to specify a functional form of the pdf *a priori*. In this article, we assume that, instead of the pdf, what is actually available is a partial set of *expectations* of observables with respect to the unknown probability measure, which may arise from experiment or other types of modelling. In this setting, information theory (in particular entropy maximization [18,19]) offers tools for constructing densities consistent with observations that are close to a given prior distribution (see [20–22] for examples in geophysical modelling, [23,24] for discussion in molecular modelling). Our algorithm for computing the parameters finds Lagrange multipliers (one for each observable) that modify the probability density, using iterative techniques like those in Agmon *et al.* [25], Haken [26], Davis & Gutiérrez [27], where each step of iteration involves ensemble simulation that reweights samples from the prior distribution appropriately. The density resulting from such an entropy maximization is then used as the basis for designing a thermostat that ensures the extended dynamical system samples the distribution and consequently exhibits the correct (long-term) statistical averages, while mildly perturbing the short-term dynamical behaviour of the system. The complexity and generality of our iterative procedure (sampling→parametrization→sampling…) means that convergence has to be verified through computer experiment.

Entropy maximization also forms the basis for closure approximations in the works of Ilg *et al.* [3] and Samaey *et al.* [28], where the result from entropy maximization is used in determining evolution equations for resolved macroscopic quantities. This approach is distinct in philosophy from the current work in which the result of entropy maximization is used to define a statistical ensemble and evolution equations for *microscopic* dynamics that are consistent with macroscopic observations.

While we motivate the method in the setting of equilibrium sampling of a fixed target distribution, we can easily apply our scheme to the case of a more general model with unknown or even undefined steady state. For this purpose, an ensemble of simulations are run in finite duration bursts, approximating the Lagrange multipliers via an iterative procedure based on the simulation history. This procedure allows entropy maximization to be performed with ensemble averages that evolve in time, providing a flexible method of nonlinear data assimilation.

The remainder of this article is organized as follows. In §2, we review the idea of a thermostat which is the key tool we employ to control the invariant distribution. Section 3 describes the combination of thermostats with the maximum entropy framework for correcting the density to reflect thermodynamic constraints. Section 4 describes the adaptive procedure. In §5, we apply and evaluate the method in the setting of a system of point vortices on the surface of a sphere, which represents a simple geophysical model with multiple statistically relevant first integrals.

## 2. Thermostats

We introduce the thermostat technique for generic Hamiltonian dynamical systems of the form

2.1 $$\frac{dy}{dt}=f(y)=B(y)\nabla H(y),\;y(t)\in D,\;B(y)=-B{\left(y\right)}^{T}\;\mathrm{and}\;H(y):D\to R,$$

possessing a divergence-free vector field^1^ ∇⋅*f*≡0. Invariance of the Hamiltonian *H* along solutions of (2.1) follows from (d/d*t*)*H*(*y*(*t*))=∇*H*⋅d*y*/d*t*=∇*H*⋅*B*∇*H*=0, due to skew-symmetry of *B*(*y*). Additional first integrals may exist, denoted by *I*_ℓ_(*y*):∇*I*_ℓ_⋅*f*≡0, ℓ=1,…,*L*.

Thermostats were introduced in molecular dynamics to model the trajectories of molecules in a fluid at constant temperature. In the common statistical mechanical framework, it is assumed that the trajectories of a system of particles in thermal equilibrium with a reservoir at constant temperature sample the canonical distribution, with smooth invariant density proportional to *exp*(−*βH*) where *β* is a parameter reciprocal to temperature scaled by Boltzmann’s constant. The trajectories of the Hamiltonian system (2.1) are restricted to a level set of energy. Hence, to model a system at constant temperature, it is necessary to perturb the vector field to make trajectories ergodic with respect to the canonical distribution. The most common way of achieving this is by adding stochastic and dissipative terms satisfying a fluctuation–dissipation relation (Langevin dynamics), which is rigorously ergodic [29].

However, DelSole [30] points out that additive stochastic forcing of trajectories leads to inaccurate dynamical quantities as autocorrelation functions are strongly perturbed. For smooth deterministic Hamiltonian dynamics, normalized velocity autocorrelation functions are of the form 1−*cτ*^2^, *c*>0 in the zero-lag limit τ→0, whereas the autocorrelation of a variable that is directly forced by white noise must take the form exp⁡(−κτ), *κ*>0 in the same limit. This implies that direct stochastic perturbation leads to autocorrelation functions that have non-zero slope and opposite curvature at zero lag [31].

An alternative approach, due to Nosé [14,15] and Hoover [32], augments the phase space with an additional thermostat variable *ξ*(*t*) (driven by a differential equation) in order to ensure that the extended dynamics on **R**^*d*^×**R** preserves an equilibrium density whose marginal on **R**^*d*^ is the target (e.g. Gibbs) density. The deterministic thermostats of Nosé and Hoover are not ergodic, but they can be combined with stochastic forcing of the thermostat variable *ξ*, leading to the so-called Nosé–Hoover–Langevin method [33]. For generic divergence-free dynamical systems, the generalized Bulgac–Kusnezov (GBK) [34,35] thermostat provides the augmented system:

2.2a dy=f(y) dt+ξεg(y) dt

and

2.2b $$d\xi =\varepsilon h(y)\;dt-\gamma \xi \;dt+\sqrt{2\gamma }\;dw,$$

where *ε*>0 and *γ*>0 are parameters, *w*(*t*) is a scalar Wiener process, and *g* and *h* are vector fields which we discuss in more detail below. Given a target density ρ(y)∝exp⁡(−A(y)), A:D→R, denote the augmented product density by $\tilde{\rho }(y,\xi )=\rho (y)\cdot \mu (\xi )$, with *μ* a univariate normal distribution with mean zero and standard deviation one. It is easily checked that $\tilde{\rho }$ is stationary under the Fokker–Planck operator associated with (2.2) provided

2.3 h(y)=∇⋅g−g⋅∇A

and provided the ‘potential’ *A* is only a function of *y* through the first integrals of *f*. The (extended) target measure $\tilde{\rho }$ is ergodic if the vector fields *f* and *g* satisfy a Hörmander condition [36]. Up to verification of the Hörmander condition, the choice of *g* is free. In the example of §5, we consider a specific form for *g*.

The parameter *ε* controls the strength of the thermostat relative to that of the vector field *f*. This affects the *rate* at which the invariant measure is sampled. It has been demonstrated in Bajars *et al.* [36,37] and Leimkuhler *et al.* [31] that GBK/NHL thermostating gives a weak perturbation of microcanonical dynamics: typical autocorrelation functions match the exact ones to leading order, i.e. have the form 1−*cτ*^2^+*O*(*τ*^3^), as τ→0.

Designing a thermostat relies on knowing the functional form of the target distribution, which may not always be available. In this article, we relax this requirement by constructing the target distribution iteratively and adaptively, based on Jaynes’ principle of least-biased ensemble prediction.

## 3. Bias correction method

Suppose that we are given a simplified dynamical model for the evolution of some projection, i.e. a ‘coarse graining’ of the phase variables (coarse-grained variables *y*(*t*)∈**R**^*d*^) which is in the form (2.1). Although the original system is complex and its details unknown, we assume that we can obtain in some way (e.g. through measurement) a collection of observations of mean values of functions of the reduced variables. That is, there are functions $C_{k}:{\text{R}}^{d}\to \text{R}$, *k*=1,2…,*K* and given values *c*_*k*_, *k*=1,2,…,*K*, such that

3.1 $$c_{k}=\langle C_{k}(y)\rangle ,\;k=1,\ldots ,K,$$

where 〈*C*_*k*_(*y*)〉 represents averaging with respect to the true, empirical invariant measure of the dynamical system. The use of a GBK thermostat (2.2) limits our implementation to observables that are functions of the conserved quantities {*H*,*I*_ℓ_,ℓ=1,…,*L*}, that is, *C*_*k*_(*y*)=*C*_*k*_(*H*(*y*),*I*_1_(*y*),…,*I*_*L*_(*y*)), *k*=1,…,*K*. This restriction is only due to the choice of thermostat, the entropy maximization has no such restriction. Our goal is to find a perturbed dynamical model for the reduced variables which (i) is compatible with the thermodynamic constraints (3.1), and (ii) mildly perturbs the dynamics compared to those of the native model.

### (a) Entropy maximization

Empirical information theory generalizes the principle of insufficient reason, by proposing the least-biased probability density consistent with a set of observations. This idea was first proposed by Jaynes [18,19] and is treated in the monographs of Haken [26], Dewar *et al.* [38] and in the context of geophysical fluids by Majda & Wang [39]. The least-biased density is defined as the probability density *ρ*(*y*) that maximizes the information entropy functional $S[\rho ]=-\int_{D}\rho (y)\log\rho (y)\;dy,$ subject to a set of constraints given by observations. When D is a compact set and there are no observations, the minimizer is the uniform density $\rho \equiv |D{|}^{-1}$. The entropy S is the unique measure of uncertainty that is positive valued, monotonically increasing as a function of uncertainty, and additive for independent random variables. With observable functions {*C*_*k*_(*y*) | *k*=1,2,…*K*} let

3.2 $$E_{\rho }C_{k}=\int_{D}C_{k}(y)\rho (y)\;dy$$

denote expectation in the (as yet undetermined) density *ρ*. Defining Lagrange multipliers *λ*_*k*_, *k*=1,…,*K*, associated with the observables *C*_*k*_, we obtain the solution *ρ* from a stationarity condition for the Euler–Lagrange functional

$$W(\rho ,\lambda_{1},\ldots \lambda_{K}):=\left[S[\hat{\rho }]-\sum_{k=1}^{K}\lambda_{k}(E_{\hat{\rho }}C_{k}(y)-c_{k})\right].$$

Conditions for a unique solution to exist are discussed in Balian [40]. When it exists, the maximum entropy solution satisfies

$$\rho (y)=\lambda_{0}\exp(-\lambda_{1}C_{1}(y)-\cdots -\lambda_{K}C_{K}(y)),$$

where *λ*_0_ is chosen to satisfy $\int_{D}\rho \;dy=1$, and *λ*_*k*_ is chosen such that $E_{\rho }C_{k}(y)=c_{k}$.

In some cases, besides the observations, we may be given prior statistical information on the process *y*(*t*). The Kullback–Leibler divergence, or relative entropy,

$$S_{\pi }[\rho (y)]=\int \rho (y)\ln\frac{\rho (y)}{\pi (y)}\;dy$$

represents a (non-symmetric) distance between measures. Note the change of sign, which makes the relative entropy more like a distance measure; we stick to this convention. It quantifies the information lost in approximating *ρ*(*y*) by *π*(*y*).

Suppose *y* is a random variable with distribution (law) *y*∼*ρ*, where *ρ* is unknown. Suppose further, that we are given a prior distribution *π*, presumed to be close to *ρ*, and a set of *K* observations (3.1). Following Jaynes [18,19], the least-biased distribution *ρ* consistent with the observations *c*_*k*_ and prior *π* solves the constrained minimization problem

$$\rho =\underset{\hat{\rho }}{\arg\;\min}\left[S_{\pi }-\lambda_{0}\left(1-\int \hat{\rho }(y)\;dy\right)-\sum_{k=0}^{K}\lambda_{k}\left(c_{k}-\int C_{k}(y)\hat{\rho }(y)\;dy\right)\right],$$

where the *λ*_*k*_ are Lagrange multipliers to enforce the condition that the expectations (3.2) agree with the observations (3.1). The solution to the variational problem is

3.3 $$\rho (y)=\lambda_{0}\exp(-\lambda_{1}C_{1}(y)-\cdots -\lambda_{K}C_{K}(y))\pi (y),$$

where the Lagrange multipliers *λ*_*k*_ are chosen consistently with the observations (3.1) and *λ*_0_ is a normalization constant so that *ρ* is a probability density function.

Calculation of the Lagrange multipliers is discussed in [25–27]. We use the following algorithm based on re-weighting. Assume we are given a sequence of samples *y*^*n*^, *n*=1,…,*N*, distributed according to a known prior distribution *π*(*y*), i.e. *y*^*n*^∼*π*. The expectation under *π*(*y*) of a function *Φ*(*y*) has the consistent and unbiased estimator

$${\hat{\Phi }}^{\pi }=\frac{1}{N}\sum_{n=1}^{N}\Phi (y^{n}).$$

Given the posterior distribution *ρ*(*y*) of the form (3.3), compute the expectation $E_{\rho }\Phi$ by re-weighting of the integral

$$E_{\rho }\Phi =\int \Phi (y)\rho (y)\;dy=\lambda_{0}\int \Phi (y)e^{-\sum_{i=1}^{K}\lambda_{i}C_{i}(y)}\pi (y)\;dy=\lambda_{0}E_{\pi }\{\Phi (y)\lambda_{0}e^{-\sum_{i=1}^{K}\lambda_{i}C_{i}(y)}\},$$

yielding an unbiased estimator for $E_{\rho }\Phi$ given by

$${\hat{\Phi }}^{\rho }=\frac{\lambda_{0}}{N}\sum_{n=1}^{N}\Phi (y^{n})e^{-\sum_{i=1}^{K}\lambda_{i}C_{i}(y^{n})}.$$

We wish to ensure that the estimators ${\hat{C}}_{k}^{\rho }$ for observables *C*_*k*_ match their empirical values *c*_*k*_, i.e.

$$c_{k}={\hat{C}}_{k}^{\rho }=\frac{\lambda_{0}}{N}\sum_{n=1}^{N}C_{k}(y^{n})e^{-\sum_{i=1}^{K}\lambda_{i}C_{i}(y^{n})},\;k=1,\ldots ,K.$$

We can use this fact to define a Newton–Raphson iteration to determine the Lagrange multipliers *λ*_*k*_. Define the residual *r* with components

$$r_{k}(\lambda )=c_{k}-\frac{\lambda_{0}}{N}\sum_{n=1}^{N}C_{k}(y^{n})e^{-\sum_{i=1}^{K}\lambda_{i}C_{i}(y^{n})},\;k=1,\ldots ,K,$$

with *λ*=(*λ*_1_,…,*λ*_*K*_) and *r*=(*r*_1_(*λ*),…,*r*_*K*_(*λ*)). Note that *λ*_0_ can be viewed as a function of *λ*_1_,*λ*_2_,…,*λ*_*K*_ chosen from the normalization condition, i.e.

$$\lambda_{0}={\left[\sum_{n=1}^{N}e^{-\sum_{j=1}^{K}\lambda_{j}C_{j}(y^{n})}\right]}^{-1}.$$

The Jacobian matrix *J*=(*J*_*kj*_) of the vector function *r* is determined as

$$J_{kj}(\lambda ):=\frac{\partial r_{k}}{\partial \lambda_{j}}=\frac{\lambda_{0}}{N}\sum_{n=1}^{N}C_{k}(y^{n})C_{j}(y^{n})e^{-\sum_{i=1}^{K}\lambda_{i}C_{i}(y^{n})}\;j,k=1,\ldots ,K.$$

The iteration then proceeds as $\lambda^{\alpha +1}\leftarrow \lambda^{\alpha }-J^{-1}(\lambda^{\alpha })r(\lambda^{\alpha })$.

## 4. Adaptive determination of Lagrange multipliers

When the observations deviate far from the prior, the Lagrange multipliers take larger values and the weights of the samples diverge, i.e. some weights become very large and others become very small. This results in a larger variance on the estimates and consequently in poorer results for the Lagrange multipliers. Moreover, the approach of the previous section excludes applications where (i) the statistical knowledge is expected to improve as the simulation progresses, (ii) the average observables are known to vary slowly with time or (iii) it is unfeasible to construct a large enough ensemble distributed in the prior. For these cases we consider using the simulation data of a small ensemble (propagated in short bursts of *M* timesteps) for updating the Lagrange multipliers for mean observation data. This results in an adaptive algorithm for obtaining the Lagrange multipliers on-the-fly during simulation. In this situation, the sample set used is drawn from a distribution closer to the target, resulting in better estimations for the residuals and Jacobian.

Let us assume that we have a (moderately sized) ensemble of initial conditions according to some distribution

4.1 $$\rho_{0}(y)=\pi (y)\exp\left(-\sum_{k=1}^{K}\lambda_{k}^{0}C_{k}(y)\right).$$

In our numerical experiments, we assume $\lambda_{k}^{0}=0$, ∀ *k* and hence *ρ*_0_=*π*, but we present the general form here nevertheless. Rather than first computing the Lagrange multipliers corresponding to observations and then running the simulation over the time interval of interest, we perform a short burst of the thermostatted simulation for *P* ensemble members, using the Lagrange multipliers $\lambda_{k}^{0}$ in the thermostat. The system states at the end of this burst, denoted by *y*^*p*^(*MΔt*) for ensemble member *p* at time *MΔt*, are used for an estimator of the observables in the distribution *ρ*_0_:

4.2 $${\hat{C}}_{k}^{\left(1\right)}=\frac{1}{P}\sum_{p=1}^{P}C_{k}(y^{p}(M\Delta t))\lambda_{0}^{1}\exp\left(\sum_{i=1}^{K}(\lambda_{i}^{0}-\lambda_{i}^{1})C_{i}(y^{p}(\Delta t))\right),\;k=1,\ldots ,K,$$

where the superscript (1) indicates that it is an estimator for a distribution with Lagrange multipliers $\lambda_{k}^{1}$. Note that the samples being distributed in *ρ*_0_ and not in *π* leads to the appearance of the Lagrange multipliers $\lambda_{k}^{0}$, this is a simple importance sampling procedure. A Newton–Raphson procedure similar to that in §3 is then used to find $\lambda_{k}^{1}$ using the estimator (4.2). The resulting multipliers are used in the second burst of thermostatted simulations, sampling $\rho_{1}(y)=\pi (y)\exp(-\sum_{k=1}^{K}\lambda_{k}^{1}C_{k}(y))$. Now, crucially, this estimator from the ensemble after two simulation bursts is combined with that at the end of the first burst. As both estimators are unbiased, we may use any weighted sum of the two (with the sum of the weights equal to one) to find another estimator. For simplicity, we weight both estimators equally. We write the combination of estimators for the general case after *L* bursts of the simulation, using the system states at the end of each previous burst, that is *y*^*p*^(ℓ*M*Δ*t*) for ℓ=1,2,…,*L*, as an estimator for the densities *ρ*_ℓ−1_ with the Lagrange multipliers $\lambda_{k}^{\ell -1}$. This estimator takes the form

4.3 $${\hat{C}}_{k}^{\left(L\right)}=\frac{1}{LP}\sum_{p=1}^{P}\sum_{\ell =1}^{L}C_{k}(y^{p}(\ell M\Delta t))\lambda_{0}^{\ell -1}\exp\left(\sum_{i=1}^{K}(\lambda_{i}^{\ell -1}-\lambda_{i}^{L})C_{j}(y^{p}(\ell M\Delta t))\right),k=1,\ldots ,K.$$

As with the non-adaptive scheme, a Newton–Raphson procedure is used to find Lagrange multipliers such that ${\hat{C}}_{k}^{\left(L\right)}=c_{k}$ with the residuals

4.4 $$r_{k}^{L}={\hat{C}}_{k}^{\left(L\right)}-c_{k}\;k=1,\ldots ,K,$$

and the gradients

4.5 $$\frac{\partial r_{k}^{m}}{\partial \lambda_{j}^{m}}=\frac{1}{mP}\sum_{p=1}^{P}\sum_{\ell =0}^{m-1}C_{k}(y_{\ell M}^{p})C_{j}(y_{\ell M}^{p})\lambda_{0}^{\ell }\exp\left(\sum_{i=1}^{K}(\lambda_{i}^{l}-\lambda_{i}^{m})C_{i}(y_{\ell M}^{p})\right),\;j,k=1,\ldots ,K.$$

By performing this iteration after each burst, and using the result for the subsequent burst, the Lagrange multipliers are computed ‘on-the-fly’ without the need for constructing a large ensemble in the prior.

Let us consider two limiting cases to justify the approach. (i) By increasing the number of ensemble members *P*, one recovers the non-adaptive scheme, where the Lagrange multipliers are found based on the initial ensemble after a single burst. On the other hand, (ii) if we have a modest ensemble *P*, it is necessary that the burst is long enough for the thermostatted dynamics with the adaptive parameters to equilibrate on this time scale. The error due to the transient of the thermostat is removed in the limit of many timesteps per burst, i.e. as M→∞. We present numerical verification of the practical use of the scheme in a specific application setting in §6c.

### (a) Adaptive algorithm

There are two important modifications to the algorithm described above that are included in the numerical implementation of this method:

— If the Lagrange multipliers change rapidly, the thermostat may require a long time to equilibrate. This requires a larger value for *M*, increasing the simulation time required before including new samples. The effect is notable at the beginning of a simulation, due to two factors: (i) the small sample size leads to inaccurate expectations for the observables, and (ii) the initial values for Lagrange multipliers may be far from their equilibrium. By limiting the rate of change of the Lagrange multipliers, these problems are circumvented.
— In equations (4.4) and (4.5), *all* previous values $\lambda_{k}^{\ell }$ are included. In a long simulation, this leads to a growing computational demand. By taking only a fixed number (*q*) of recent steps the cost is reduced. In the case that the initial samples cannot accurately be drawn from the prior, this has the further advantage that these inaccuracies are eventually forgotten.

The resulting method is summarized in algorithm 1.


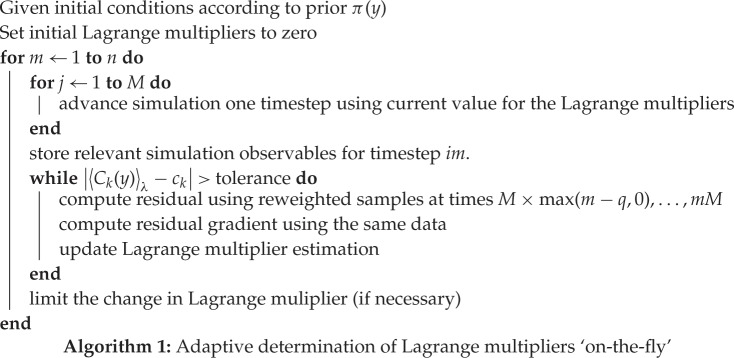


## 5. Application to reduced modelling of point vortices

Here we apply the least-biased correction method to a system of point vortices on the sphere, which has a Poisson structure and multiple conserved quantities, including total energy and angular momentum and a set of Casimirs.

### (a) Point vortex system

Point vortices represent singular solutions to two-dimensional, incompressible fluid flow in which each vortex, positive or negative, is centred at a specified position. Point vortices have been studied extensively in [41–43]. We work on a spherical domain [44–48], viewing the vortex position as a vector in **R**^3^ with unit norm. The equations of motion have a Lie–Poisson structure

5.1 $$\Gamma_{i}{\dot{\text{x}}}_{i}={\text{x}}_{i}\times \nabla_{{\text{x}}_{i}}H\;i=1,2,\ldots ,M,$$

where the Hamiltonian is given by

$$H=-\sum_{i=1}^{M}\sum_{j=1}^{i-1}\frac{\Gamma_{i}\Gamma_{j}}{4\pi }\ln(2-2{\text{x}}_{i}\cdot {\text{x}}_{j})$$

and each *Γ*_*i*_ represents the circulation of a single point vortex.

By introducing $\text{ y}={\left({\text{ x}}_{1}^{T},{\text{ x}}_{2}^{T},\ldots ,{\text{ x}}_{M}^{T}\right)}^{T}$, equation (5.1) has the form (2.1) with the block-diagonal structure matrix

$$B(\text{y})=\left[\begin{array}{cccc} \Gamma_{1}^{-1}{\hat{\text{x}}}_{1} & & & \\ & \Gamma_{2}^{-1}{\hat{\text{x}}}_{2} & & \\ & & \ddots & \\ & & & \Gamma_{M}^{-1}{\hat{\text{x}}}_{M} \end{array}\right],$$

where ${\hat{\text{ x}}}_{i}$ is the 3×3 skew-matrix satisfying ${\hat{\text{ x}}}_{i}\text{ a}:={\text{ x}}_{i}\times \text{ a}$, for all ***a***∈**R**^3^. The Poisson bracket for the system is given equivalently by

$$\left\{F,G\right\}=\sum_{i=1}^{M}\frac{1}{\Gamma_{i}}\nabla_{{\text{x}}_{i}}F\cdot ({\text{x}}_{i}\times \nabla_{{\text{x}}_{i}}G)\;\text{or}\;\left\{F,G\right\}=\nabla F{\left(\text{y}\right)}^{T}B(\text{y})\nabla G(\text{y}).$$

This Poisson structure generalizes the rigid body Poisson structure and also occurs in ferromagnetic spin lattices [49–51] and elastic rods (e.g. [52]).

The vortex positions are defined in Cartesian coordinates, but initial positions ***x***_*i*_(0) are chosen on the sphere. Because each |***x***_*i*_| is a Casimir of the Poisson bracket, the vortices remain on the sphere. Furthermore, the rotational symmetry of the sphere gives rise to three Noether momenta, which are expressed by the angular momentum vector

5.2 $$\text{J}=\sum_{i=1}^{M}\Gamma_{i}{\text{x}}_{i}.$$

The GBK thermostat (2.2) is only applicable to divergence free systems ∇⋅*f*≡0. It is straightforward to check that this condition holds for the spherical point vortex model.

A numerical integrator for the point vortex system is constructed as in Myerscough & Frank [48] based on splitting the differential equations into integrable subproblems (see related ideas in [53,54]). The backward error analysis of symplectic integrators has an analogous development for Poisson systems, implying approximate conservation of the Hamiltonian [8]. The Casimirs (|***x***_*i*_|≡1) and the momentum $\text{ J}=\sum \Gamma_{i}{\text{ x}}_{i}$ are exactly preserved by the integrator.

### (b) Thermostat perturbation vector

The choice of the perturbation vector field *g* in (2.2) is flexible, but the vector fields *f* and *g* should satisfy a Hörmander condition [36]. It is vital that the Casimirs should be respected by the perturbation vector field. Double-bracket dissipation [55] preserves Casimirs of the original system and is thus an ideal candidate for *g*:

5.3 $${\tilde{g}}_{i}({\text{x}}_{i})=\sum_{j\neq i}{\text{x}}_{i}\times {\text{x}}_{i}\times \frac{\Gamma_{j}}{4\pi }\frac{{\text{x}}_{j}}{1-{\text{x}}_{i}\cdot {\text{x}}_{j}}.$$

This ‘double-bracket thermostat’ also has the advantage that it can be split into pairwise interactions along with the original dynamics *f*, simplifying computation. The denominator in (5.3) leads to a stiff differential equation when like-signed vortices approach one another, restricting the step size of an explicit splitting method. For this reason, we use a slightly modified scheme (but still Casimir-preserving) defined by

5.4 $$g_{i}({\text{x}}_{i})=\sum_{j\neq i}{\text{x}}_{i}\times {\text{x}}_{i}\times \frac{\Gamma_{j}}{4\pi }{\text{x}}_{j}.$$

Details of the numerical integration of these dynamics may be found in appendix A.

The thermostat (2.2) is designed to sample a target density *ρ*(*y*)∝*e*^−*A*(*y*)^ on the phase space of *y*. The thermostat variable *ξ* is normally distributed, yielding the extended distribution $\rho \propto e^{-A(y)-\frac{1}{2}\xi^{2}}$. The perturbation vector field *g* must additionally ensure that the thermostatted system is ergodic in the target density. Because the target measure is positive for all open sets on the phase space, hypoellipticity of the Fokker–Planck equation associated with (2.2) is sufficient to prove uniqueness of the invariant measure [56]. Hypoellipticity follows from Hörmander’s controllability condition [57]. The condition has been tailored to GBK thermostats in Bajars *et al.* [36], but it is difficult to verify in practice. Here we verify using simulation that single trajectories have statistics that agree with the target distribution.

### (c) Maximum entropy model

To apply the methods proposed in §3 in the setting of a reduced model for point vortices, we use point vortices distributed evenly over the surface of the sphere as the prior *π*. The use of a thermostat for the bias correction limits us to using observables that are function of the first integrals. Furthermore, we assume the angular momentum has no directional preference, a choice we motivate in the numerical comparisons of §6. In that case, only the magnitude |***J***| of the momentum vector needs to be considered.

To accurately reproduce statistics from a full model with a moderate number of point vortices, it is necessary to modify the canonical density with a term quadratic in the Hamiltonian, that is, a density of the form $\rho (y)\propto \exp(-\beta H(y)-\gamma H{\left(y\right)}^{2})$ [16]. Motivated by Dubinkina *et al.* [16], we choose observations that include linear and quadratic functions of *H* and |***J***|^2^.

We consider the following set of observables:

5.5 $$C_{1}=H,\;C_{2}=|\text{J}{|}^{2},\;C_{3}=H^{2},\;C_{4}=|\text{J}{|}^{4}\;\mathrm{and}\;C_{5}=H|\text{J}{|}^{2}$$

and denote the corresponding Lagrange multipliers by *β*_*H*_, *β*_*J*_, *γ*_*H*_, *γ*_*J*_ and *γ*_*HJ*_.

The least-biased density consistent with observations of the $EC_{k}$ is given by

5.6 $$\tilde{\rho }(H)=e^{-\beta_{H}H-\beta_{J}|\text{J}{|}^{2}-\gamma_{H}H^{2}-\gamma_{J}|\text{J}{|}^{4}-\gamma_{HJ}H|\text{J}{|}^{2}},$$

where the Lagrange multipliers are to be found via the procedure detailed in §3a or that of §4.

## 6. Numerical comparison

Here we apply algorithm 1 to a reduced model of point vortices similar to the configuration used in Bühler [58] and Dubinkina *et al.* [16]. We distinguish between three models. The *full model* consists of a system (5.1) of *M*_full_=288 point vortices, of which eight strong vortices of circulation *Γ*_*j*_=±1 and 280 weak vortices of circulation $\Gamma_{j}=\pm \frac{1}{5}$. Both strong and weak classes comprised equal numbers of positively and negatively oriented point vortices. The *reduced model* consists of (5.1) with just *M*=8 strong vortices. Finally, the *corrected model* consists of a thermostatted system (2.2) with unperturbed vector field *f* given by (5.1) for *M*=8 strong vortices, perturbation vector field *g* given by (5.4), and equilibrium measure defined by the least-biased density (5.6). Additionally, we compare with Metropolis–Hastings samples from the least-biased density (5.6) to help distinguish between errors incurred due to the maximum-entropy model and those due to sampling bias of the (discretized) thermostatted dynamics.

We run five long simulations of the full model with total energies chosen from the set *H*_full_∈{−2,−1,0,1,2}. The total angular momentum vector is fixed at ***J***_full_=**0** such that there is no directional preference for the momentum of the reduced model embedded in the full model. For each run, we determine the time averages of the observables (5.5) for the subset of strong vortices. When computing the Hamiltonian *H* of this subsystem, we include only the internal coupling between strong vortices. The time averages are tabulated in table 1.

**Table 1.** Full model observations and (in parentheses) corrected values of first integrals.

	〈*H*〉	〈|*J*|^2^〉	〈*H*^2^〉	〈|*J*|^4^〉	〈*H*|*J*|^2^〉
*H*_full_=−2	−0.33 (−0.38)	4.59 (4.45)	0.22 (0.23)	−0.63 (−0.98)	34.58 (31.45)
*H*_full_=−1	−0.11 (−0.18)	4.78 (4.68)	0.10 (0.12)	0.38 (−0.01)	37.55 (35.88)
*H*_full_=0	0.02 (−0.04)	4.63 (4.56)	0.08 (0.08)	0.90 (0.60)	35.26 (34.30)
*H*_full_=1	0.17 (0.15)	4.74 (4.75)	0.13 (0.12)	1.73 (1.61)	37.76 (37.44)
*H*_full_=2	0.31 (0.28)	4.87 (5.00)	0.22 (0.21)	2.49 (2.46)	39.26 (41.74)

Using these averages, we compute the Lagrange multipliers using the algorithm described in §4 with prior distribution *π* taken to be uniform on the sphere. The Lagrange multipliers are also recorded in table 2. The magnitude of *γ*_{*H*,*J*,*HJ*}_ indicates that including these observables impacts the resulting density. In other words, all observables add information to the least-biased density, with the possible exception of the lowest energy case with *H*_full_=−2.

**Table 2.** Lagrange multipliers for each energy level.

	*β*_*H*_	*β*_*J*_	*γ*_*H*_	*γ*_*J*_	*γ*_*HJ*_
*H*_full_=−2	5.98	−0.20	0.69	0.41×10^−3^	−0.04
*H*_full_=−1	2.89	−0.03	2.67	9.77×10^−3^	−0.33
*H*_full_=0	−0.76	0.20	3.38	9.97×10^−3^	−0.37
*H*_full_=1	−3.54	0.37	4.29	15.31×10^−3^	−0.54
*H*_full_=2	−6.42	0.53	4.45	14.05×10^−3^	−0.51

We then run simulations of the corrected model using the computed parameters. Table 1 also records expectations from the thermostat-corrected model.

By analogy with canonical statistical mechanics, we may think of the weak vortices that are ignored in the reduced model as forming a reservoir with which our reduced model exchanges energy and angular momentum. Experience with canonical statistical mechanics of point vortices in the plane [16,58] suggests that for small reservoir sizes, the canonical distribution must be modified with higher order terms to agree with the full system statistics. Table 3 gives the multipliers for different numbers *M*_full_ of vortices in the full system, confirming that those corresponding to *γ*_*H*_, *γ*_*J*_ and *γ*_*HJ*_ are more significant for smaller *M*_full_; as the number of weak vortices grows, the strong vortex system approaches the canonical density.

**Table 3.** Lagrange multipliers as a function of *M*_full_, all for *H*_full_=0.

	*β*_*H*_	*β*_*J*_	*γ*_*H*_	*γ*_*J*_	*γ*_*HJ*_
*M*_full_=36	−1.51	1.35	27.75	117.15×10^−3^	−3.07
*M*_full_=72	−4.27	0.82	8.71	37.79×10^−3^	−1.12
*M*_full_=144	−0.97	0.32	6.70	25.80×10^−3^	−0.82
*M*_full_=288	−0.76	0.20	3.38	9.97×10^−3^	−0.37
*M*_full_=576	−1.09	0.13	0.87	3.08×10^−3^	−0.10

**Remark 6.1**

The form of the distribution (5.6) implies there is a non-zero probability for any configuration of the strong vortex system with finite energy and momentum. When considering the strong vortex system in contact with a reservoir of weak vortices, its energy is bounded by the energy in the total system plus or minus how much energy can be stored in the reservoir and in the interactions between strong and weak vortices. For the momentum, a similar condition holds, but here there are no interaction terms. If it is not possible for the reservoir to add or subtract sufficient energy from the reservoir, it will not be possible for the subsystem of strong vortices to attain all possible configurations with non-zero probability.

For the energy, it is readily verified that as long as the reservoir contains three vortices not all of equal sign, the reservoir may supply or remove an arbitrary amount of energy, as close of approaches of two like-signed (respectively, opposite-signed) vortices produces arbitrarily large-positive (respectively, negative) energies.

For the momentum, the result is more interesting. The system of *M* strong vortices, all with strength ±*Γ*_strong_, may exist in configurations with an angular momentum $\sup|{\text{ J}}_{\mathrm{red}.}|=M\Gamma_{\mathrm{strong}}$. Note the supremum, as the configuration with exactly $\sup|{\text{ J}}_{\mathrm{red}.}|=M\Gamma_{\mathrm{strong}}$ will have infinite energy. For the reservoir, it holds that $\sup|{\text{ J}}_{\mathrm{weak}}|\leq (N-M)\Gamma_{\mathrm{weak}}$. For the the reservoir to supply sufficient angular momentum, it is necessary that

$$M\Gamma_{\mathrm{strong}}\leq (M_{\mathrm{full}}-M)\Gamma_{\mathrm{weak}}\;\Leftrightarrow \;\frac{M_{\mathrm{full}}-M}{M}\geq \frac{\Gamma_{\mathrm{strong}}}{\Gamma_{\mathrm{weak}}}.$$

In the thermal bath simulations discussed in this section *M*=8 and *Γ*_strong_/*Γ*_weak_=5, this means *M*_full_ should satisfy *M*_full_≥48. The smallest system considered (*M*_full_=36) does not, explaining its eccentric parameter values in table 3.

### (a) Equilibrium results

**Figure 1.**
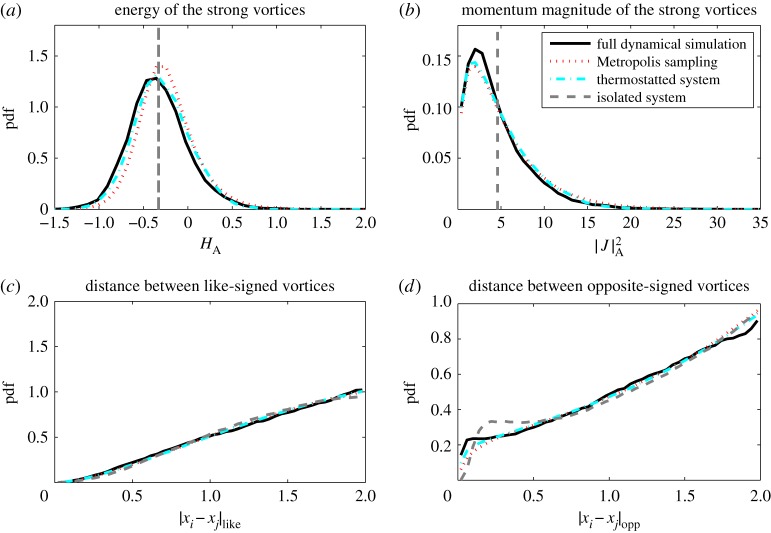
Histograms for *H*_full_≈−2. (*a*,*b*) Compare strong vortex energy and angular momentum, respectively. Panels (*c*,*d*) Compares the distance between like (resp. opposite) signed strong vortices. The parameters are specified in the text. (Online version in colour.)

**Figure 2.**
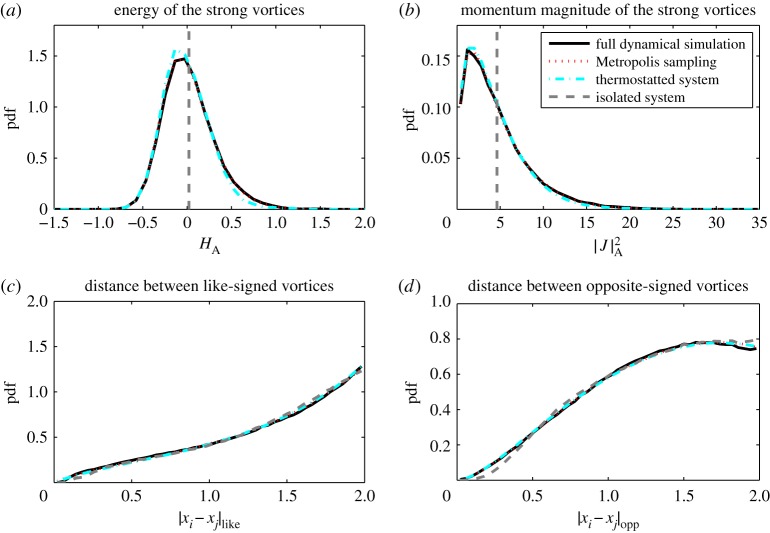
Histograms for *H*_full_≈0. Panel layouts same as figure 1. (Online version in colour.)

**Figure 3.**
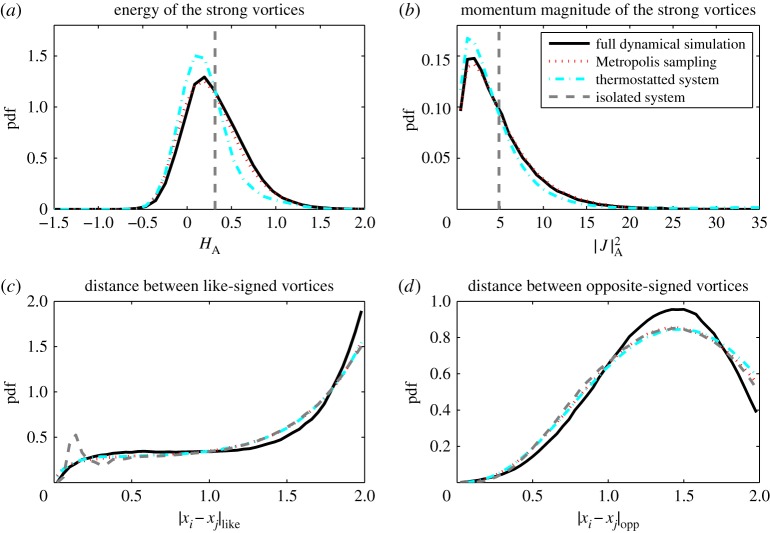
Histograms for *H*_full_≈2. Panel layouts same as figure 1. (Online version in colour.)

In this section, we compare statistical properties of the corrected model with those of the full and reduced models. In figures 1–3, we show histograms of a number of solution features for the eight vortex model: the distributions of *H* and |***J***|, as well as typical distances between like- and opposite-signed vortices, a metric also used by Bühler [58]. In each histogram, the statistics corresponding to the strong vortices in the full model, the reduced model, thermostat-corrected reduced model and Metropolis–Hastings samples are displayed. Figures 1–3 correspond to approximate total energies *H*_full_≈−2, 0 and 2, respectively.

The full and reduced model simulations are performed with a timestep of 5×10^−3^ and run up to *T*=5×10^4^, taking 10^5^ samples spaced evenly in time. For the Metropolis–Hastings method, we use 10^6^ samples. The same figures also show results from the thermostatted system (dashed-dotted lines), run with a timestep of 10^−3^ up to *T*=10^6^, taking 10^6^ samples. The parameters in (2.2) were set to be *ϵ*=10 and *γ*=0.1. These results confirm that the thermostatted system samples the least-biased density closely. The complete simulation trajectories are available in the electronic supplementary material.

The reduced model is Hamiltonian and the Poisson integrator ensures that the energy is conserved with a standard deviation of order 10^−3^ and the angular momentum constant to machine precision. Both cases correspond to approximate delta-distributions in the upper histograms in figures 1–3. Note that due to the high skewness of the distribution for |***J***|, the observed mean differs significantly from the median and mode, implying some ambiguity in choosing the angular momentum for an appropriate initial condition for the reduced model.

A simple Hamiltonian-reduced model is incapable of sampling the energy and angular momentum spectra, as these are first integrals, hence the reduced model shows significant bias in statistics such as vortex separation. On the other hand, the thermostat-corrected model faithfully samples the least-biased probability density, as indicated by the good agreement in the histograms of the corrected model and Metropolis–Hastings samples. The least-biased density approximates the strong-vortex statistics well, particularly in the negative to moderate total energy regime. At large positive total energies, the strong vortex energy and angular momentum distributions are still well represented by the least-biased PDF, but some bias in the vortex separations can be observed. The closeness of the thermostat results to those from the Metropolis–Hastings sampling indicate the error lies in the choice of least-biased density, not in the thermostat sampling.

### (b) Dynamic consistency

The results in the previous section confirm that the thermostatted simulations lead to equilibrium distributions of observables *H* and |***J***| similar to those of the full system. In this section, we address the issue of the degree to which our equilibrium correction mechanism disturbs dynamics, as encoded in autocorrelation functions and diffusivity. Diffusivity was considered by Chavanis [59] for a system of identical point vortices and by Cotter & Pavliotis [60] for a wide array of problems with scale separation. We emphasize that the values of the thermostat parameters *ε* and *γ* have no impact on the equilibrium statistics presented in the previous section, and only affect the rate at which the least-biased PDF is sampled. Parameters with a larger deviation from the unperturbed dynamics lead to faster equilibration of the distribution, giving rise to a modelling choice.

#### (i) Autocorrelation functions

Given a sequence of *L* equally spaced observation times *t*_*i*_∈[0,*T*] for *i*∈[0,*L*], and the values of the relevant observable (in our case vortex position) *u*_*i*_=*u*(*t*_*i*_) at those times, the discrete autocorrelation function is defined by

$$\nu_{i}^{u}=\frac{1}{L-i}\sum_{j=i}^{L}u(t_{j})u(t_{j-i}).$$

A normalized autocorrelation function ${\hat{\nu }}^{u}$ is given by dividing each $\nu_{i}^{u}$ by $\nu_{0}^{u}$, i.e. ${\hat{\nu }}_{i}^{u}=\nu_{i}^{u}/\nu_{0}^{u}$.

We average the autocorrelation function over all 3*M* (strong) vortex coordinates {*x*_*m*_,*y*_*m*_,*z*_*m*_}_*m*=1…*M*_. Three symmetries in the problem justify this averaging: the vortex numbering is arbitrary; the choice of reference frame is arbitrary and the sign of the vortices appears in the dynamics as a reversal of time, to which the autocorrelation is insensitive. Additionally, the observables *H* and |***J***| are isotropic.

Furthermore, we ensure that the phase space is well sampled by averaging the autocorrelation functions over an ensemble of *P* solutions. We then find the average autocorrelation function

6.1 $$\nu_{i}=\frac{1}{3MP(L-i)}\sum_{p=1}^{P}\sum_{m=1}^{M}\sum_{j=i}^{L}x_{m}^{p}(t_{j})x_{m}^{p}(t_{j-i})+y_{m}^{p}(t_{j})y_{m}^{p}(t_{j-i})+z_{m}^{p}(t_{j})z_{m}^{p}(t_{j-i}),$$

where a superscript *p* represents the solution from ensemble member *p*. The normalized average autocorrelation function is given by

6.2 $${\hat{\nu }}_{i}=\frac{1}{MP(L-i)}\sum_{p=1}^{P}\sum_{m=1}^{M}\sum_{j=i}^{L}{\text{x}}_{m}^{p}(t_{j})\cdot {\text{x}}_{m}^{p}(t_{j-i}),$$

where we have used that the Casimirs *C*_*i*_=***x***_*i*_(*t*)⋅***x***_*i*_(*t*)=1 ∀ *i*,*t* are conserved exactly, also in the discretized equations.

**Figure 4.**
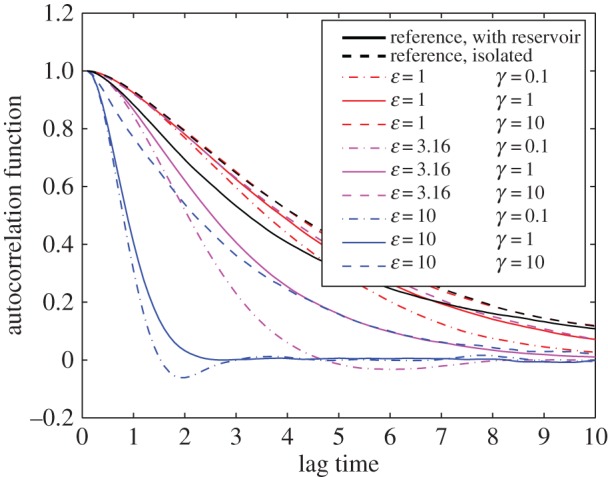
Comparison of autocorrelation of the vortex coordinates. The bold lines are two reference cases: the full model (solid) and the reduced model (dashed). The thin lines indicate autocorrelation functions of the thermostat-corrected model for indicated values of parameters *ε* and *γ*. (Online version in colour.)

In figure 4, we compare autocorrelation functions for the strong vortices in the full and reduced models as well as for the thermostat-corrected model over a range of parameters *ε* and *γ*. The thick solid black line represents the result for an (unthermostatted) system in contact with 280 weak ($\Gamma_{B}=\pm \frac{1}{5}$) vortices, with a total energy *H*_full_=0. The results present the average over an ensemble of 1000 runs. For each simulation, the initial placement of each strong vortex was taken uniformly over the sphere and the weak vortices were placed such that the full system satisfied *H*_full_=0 and ***J***_full_=0. The thick dashed black line represents the results for an ensemble of simulations of the isolated system, with everything else unchanged. The other lines represent results for thermostatted simulations using the Lagrange multipliers as given in table 2 for the case of *H*=0. As expected, when the thermostat is weak the autocorrelation functions are similar to those of the isolated system. The stronger thermostat parameters presented here match the autocorrelation well for a short-time period, but all result in excessive decorrelation for longer lag times. In the following section on the diffusivity constant, we discuss the performance of different parameter values in more detail.

#### (ii) Diffusivity

For general multiscale dynamical systems with a separation of slow and fast dynamics, it is often desirable to model fast forces by a diffusion process, resulting in stochastic differential equation of the form [61,62]

dX=f(X) dt+K(X) dW,

where *f* represents the slow dynamics, *W* is a Wiener process and *K* is the diffusivity. The value of the diffusivity *K* can be estimated by sampling solutions to the original, multiscale, problem and applying Kubo’s formula

$$K_{\Delta \tau }=\frac{\left\langle \Delta X\Delta X\right\rangle }{2\Delta \tau },$$

where Δ*X* represents displacement during the sampling interval Δ*τ*. Choosing the correct sampling interval is a notorious problem; for a comparison, see [61].

If we take the average diffusivity for each vortex coordinate, we find

$$K_{\Delta \tau }=\frac{1}{6M\Delta \tau }\sum_{m=1}^{M}\left\langle \Delta {\text{x}}_{m}\cdot \Delta {\text{x}}_{m}\right\rangle .$$

We assume the observations are given at the same times *t*_*i*_ as before and that the sampling time is an integer multiple of the observation interval, i.e. Δ*τ*=*k*(*T*/*L*). With an ensemble of *P* simulations the diffusivity estimator would then be

$$\begin{array}{rl} K_{\Delta \tau } & =\frac{1}{6MP\Delta \tau }\sum_{p=1}^{P}\sum_{m=1}^{M}({\text{x}}_{m}^{p}(\Delta \tau )-{\text{x}}_{m}^{p}(0))\cdot ({\text{x}}_{m}^{p}(\Delta \tau )-{\text{x}}_{m}^{p}(0))\\ & =\frac{1}{6MP\Delta \tau }\sum_{p=1}^{P}\sum_{m=1}^{M}2-2{\text{x}}_{m}^{p}(0)\cdot {\text{x}}_{m}^{p}(\Delta \tau )\\ & =\frac{1}{3\Delta \tau }-\frac{1}{3MP\Delta \tau }\sum_{p=1}^{P}\sum_{m=1}^{M}{\text{x}}_{m}^{p}(0)\cdot {\text{x}}_{m}^{p}(\Delta \tau ), \end{array}$$

where again a superscript *p* denotes the solution from ensemble member *p*. Averaging over all time series data yields the estimator:

$$K_{\Delta \tau }=\frac{1}{3\Delta \tau }-\frac{1}{3MP(L-k)\Delta \tau }\sum_{p=1}^{P}\sum_{j=1}^{L-k}\sum_{m=1}^{M}{\text{x}}_{m}^{p}(t_{j})\cdot {\text{x}}_{m}^{p}(t_{j}+\Delta \tau )=\frac{1-{\hat{\nu }}_{k}}{3\Delta \tau },$$

with Δ*τ*=*k*(*T*/*L*)=*t*_*k*_. This *shift-averaged estimator* incorporates all possible time intervals of length Δ*τ* available from the data; it is shown by Cotter & Pavliotis [60] to improve the quality of the estimator.

**Figure 5.**
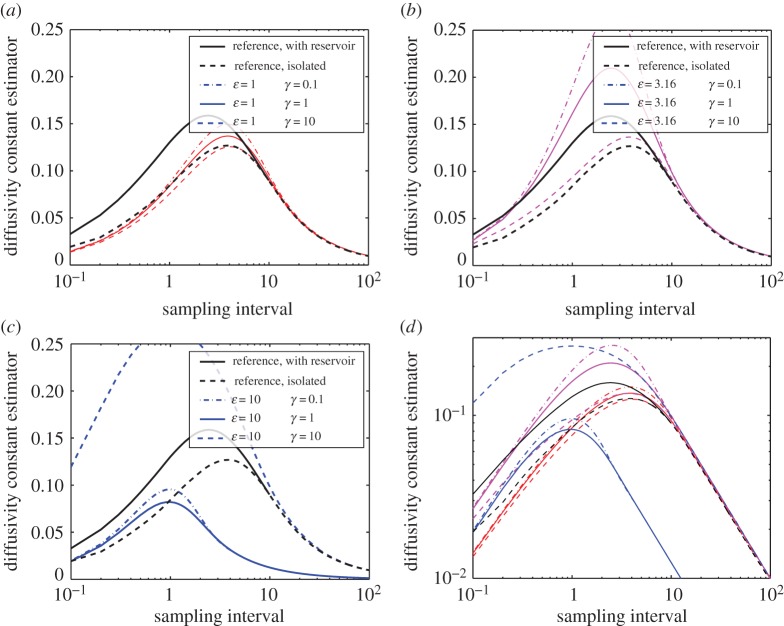
Comparison of average diffusivity constant as a function of sampling intervals. In all figures, the bold lines indicate two reference cases: the full model (solid) and the reduced model (dashed). (*a*–*c*) Thermostatted simulation results for *ϵ* equal to 10^0^, 10^0.5^ and 10^1^, respectively. The value of *γ* is represented by dashed-dotted (10^−1^), solid (10^0^) or dashed (10^1^) lines. A combined log–log plot of all parameter values is given in (*d*). (Online version in colour.)

Figure 5 presents the diffusivity constant as a function of the interval time Δ*τ* for different parameter values for *ε* and *γ*. The results are taken from the same simulations used for the autocorrelation functions, as described in the section.

For *ε* small, the thermostat perturbation is weak, and both autocorrelation functions and diffusivity approach those of the reduced model with constant *H*, ***J***. Also, the autocorrelations are insensitive to the parameter *γ* in this regime. For values of *ε*<1, the autocorrelations and diffusivities become indistinguishable from those of the isolated model. Hence even though the dynamics samples the least-biased density on long time scales, its short-time dynamics is similar to an unperturbed model. For moderate *ε*, dependence on *γ* becomes more pronounced, and a diffusivity closer to that of the full model can be achieved. For even larger values of *ε*, the diffusivity becomes much more sensitive to the value of *γ*, as indicated in figure 5*c*.

Figure 5*d* has been included to illustrate two important properties by plotting the diffusion parameter against the sampling interval on a log–log scale. First, for large sampling interval the estimator shows an inverse linear tendency. This corresponds simply to the decorrelation of the vortex dynamics. Second, as the sampling interval goes to zero, the estimator of the diffusivity constant shows polynomial behaviour. This is in agreement with known results for the GBK thermostat [63] and is an improvement on Langevin thermostats, which tend to a constant value for short sampling intervals, in disagreement with a deterministic reference [30]. See also the discussion in §2.

### (c) Adaptive determination of multipliers

In this section, we apply the scheme of §4 to the point vortex system. Consider the same reduced point vortex model of eight vortices with *Γ*=±1. We perform a thermostatted simulation of this system, which we feed with observation data taken from a simulation of the 288-vortex system including the weak vortices that form the thermal bath. The observation from simulation with the thermal bath may be replaced by observation from another source–if available–such as empirical observations or a prediction of the trend for the observables. In particular, we use the observed data in three different ways for computing the Lagrange multipliers that determine the equilibrium distribution used by the thermostat:

(i) The first approach mirrors the non-adaptive method of §3. We use the *long time averages* from equilibrated thermal bath simulation. The equilibrium distribution varies during the thermostatted simulation, but during the whole simulation it converges to the same equilibrium density as before (like those denoted in table 2).
(ii) The second approach does not assume long time averages are available *a priori*. Instead, the *running mean* from the thermal bath simulation is used. This means the iterative procedure (4.4) drives the system towards a varying target during simulation. Eventually, the target approaches the long time average used above.
(iii) In the final implementation, we relax the notion of statistical equilibrium for the strong system and consider the statistical state to be slowly varying. This is implemented by using a *time-local averaged* observations from the thermal bath simulations. As a result, there is no stationary equilibrium density.

For testing the adaptive scheme, we only consider the thermal bath simulations where *H*_full_=0. Also, we consider just two observables *C*_1_=*H* and *C*_2_=|***J***|^2^, associated with the Lagrange multipliers *β*_*H*_ and *β*_*J*_. Including more observables is possible, but the Newton–Raphson iteration is sensitive to noise in the estimators, requiring a larger ensemble. We initialize with an ensemble of *P*=100 initial conditions drawn from the uniform prior, applying algorithm §4a The timestep in the thermostatted simulation is chosen as 1×10^−2^ and the method described in §4 for updating the Lagrange multipliers is applied every time unit, i.e. *M*=100. Between subsequent updates of the multipliers, the maximum difference is limited by |Δ*λ*_*k*_|≤0.1. When using equilibrium statistics, this limit only affects the beginning of the simulation, when the small sample size used leads to a large variance in the estimators.

We present the results for the three different uses of the observed data. The complete simulation trajectories are available in the electronic supplementary material.

**Figure 6.**
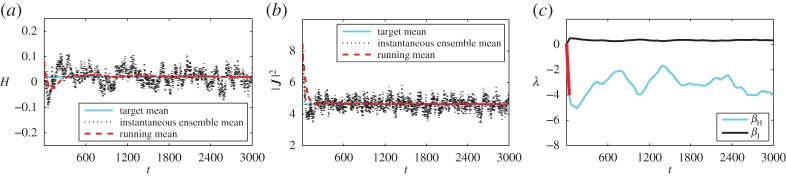
Results when using *long-time mean* observations as a target while adaptively determining the Lagrange multipliers. Target observations for Hamiltonian (*a*) and momentum magnitude (*b*) are overlaid with the instantaneous ensemble mean (black dotted line)and the running ensemble mean (red solid line) from simulation. (*c*) Lagrange multipliers, the red dots indicate time steps at which their rate of change was limited. (Online version in colour.)

**Figure 7.**
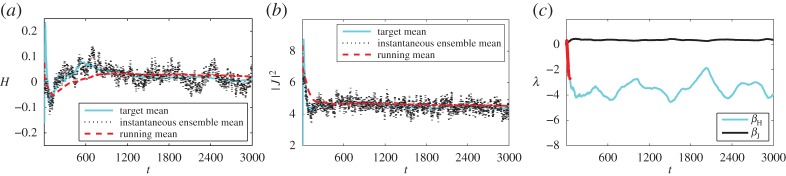
Results when using *running mean* observations as a target while adaptively determining the Lagrange multipliers. (Online version in colour.)

**Figure 8.**
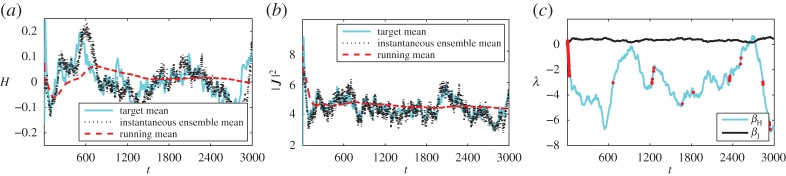
Results when using *time-local averaged* observations as a target while adaptively determining the Lagrange multipliers. (Online version in colour.)

(i) In figure 6, the long time mean is taken and used throughout.
(ii) In figure 7, the running mean is used. This reflects the situation where we have no *a priori* knowledge of the observations, and are continuously feeding new real-time data into the simulation.
(iii) In figure 8, a time-localized average of the observable is used. The averaging has a time-scale of a 100 time units.

In each of the figures, panel (*a*) shows the target mean for the energy solid cyan (light), the instantaneous ensemble mean in black dots and the running mean of the ensemble in dashed red (dark). Panel (*b*) shows the same means for the momentum magnitude and (*c*) shows the Lagrange multipliers for the energy (cyan, light) and momentum (black). Superimposed on the Lagrange multiplier for the energy is an indicator (red, darker dots) showing when the rate of change in the estimate for the Lagrange multiplier has been limited.

When using either a long time mean observation or a running mean observation, the simulation results tend towards the correct long-time averages. When using time-local averages the simulation averages appear to tend towards a similar value. In all three cases, the instantaneous ensemble mean remains close to the (moving) target for both energy and momentum. This is especially notable for the third case, where the target varies over time, but the simulation ensemble mean follows closely, with only a little lag.

The inaccuracies during the first approximately 100 time units indicate that the prior does not match the observed state well. This results in the (negative) growth of *β*_*H*_ being limited briefly at the beginning of each simulation. Subsequently, both Lagrange multipliers appear to oscillate irregularly about some mean value for the first two cases. In the case of a shifting target, the Lagrange multipliers vary in time more erratically, resulting in the limiter being active for a few brief periods of the simulation.

## 7. Conclusion

We have proposed a thermostat-based method for perturbing trajectories of numerical simulations to correct for stationary or time-dependent thermodynamic observations. We have also described an adaptive procedure that uses the thermostatted simulation data in finding the least-biased density, removing the need to compute the density *a priori*.

Our numerical experiments with the vortex model confirm that the distributions of the observed energy *H* and angular momentum magnitude |***J***| can be well approximated using the thermostat technique. Other equilibrium metrics such as the distribution of distances between like- and opposite-signed vortices are also in agreement across a range of total energy values of the full system, although some discrepancies occur at large positive energies.

We also investigated the degree to which correction of trajectories for expectations may affect dynamical information in the form of autocorrelation functions and diffusivity. By decreasing the perturbation parameter *ε* of the thermostat, the autocorrelation functions of the unperturbed, reduced system may be precisely recovered. As *ε* is increased, one may increase the diffusivity to values that agree with the full system. This is consistent with results reported in Frank & Gottwald [63], in the context of molecular dynamics where it was shown that the GBK thermostat used here approaches Langevin dynamics in the limit of large stochastic forcing.

## Supplementary Material

high_e1_d-1_HJ_Lagrange_long.mat

## Supplementary Material

higher_e1_d-1_HJ_Lagrange_long.mat

## Supplementary Material

highest_e1_d-1_HJ_Lagrange_long.mat

## Supplementary Material

low_e1_d-1_HJ_Lagrange_long.mat

## Supplementary Material

lower_e1_d-1_HJ_Lagrange_long.mat

## Supplementary Material

lowest_e1_d-1_HJ_Lagrange_long.mat

## Supplementary Material

neutral_e1_d-1_HJ_Lagrange_long.mat

## Supplementary Material

Observation-based-correction.zip

## Footnotes

1 The latter condition is automatic for systems (2.1) with constant *B*. Strictly speaking, the approach described here is applicable to any system with divergence-free vector field ∇⋅*f*≡0 possessing one or more first integrals.

## Data accessibility

The code for thermostated simulation of the point vortex system can be found at https://www.dropbox.com/sh/ekhj4ohtetj35ri/AABNMpHki63cn16hpnaQ.OYa?dl=0.

## Appendix A. Time integration of the thermostatted system

In this appendix, we detail the method of integration for the perturbation dynamics and thermostat variable.

The unperturbed dynamics are formed by the combination of pairwise interactions:

A 1 $$f(\text{y})=B(\text{y})\nabla_{\text{y}}H(\text{y})=-B(\text{y})\sum_{i=1}^{M}\sum_{j=1}^{i-1}\nabla_{\text{y}}\frac{\Gamma_{i}\Gamma_{j}}{4\pi }\ln(2-2{\text{x}}_{i}\cdot {\text{x}}_{j})=\sum_{i<j}f_{ij}.$$

This fact is exploited in our earlier work [48] to construct a splitting method for integration of the point vortex system with favourable (geometric) properties. The modified double bracket thermostat is also a combination of pairwise vortex interactions. Hence it is natural to apply the same splitting to *g* as was applied to *f*. In fact, the pairwise thermostat interaction can be executed along with the unperturbed dynamics. Let us denote by $\phi_{\Delta t}^{i,j}$ the time Δ*t* flow map associated with *f*_*ij*_. Similarly, we define by $\gamma_{\Delta t}^{i,j}$ the time Δ*t* flow map associated with the perturbation dynamics of a vortex pair (*i*,*j*). The flow map of the dynamics of the thermostat variable *ξ* is represented by *χ*_Δ*t*_. A symmetric composition of these flows is given by

$$\Phi_{\Delta t}=\prod_{(i,j)\in C}(\phi_{\Delta t/2}^{i,j}\circ \gamma_{\Delta t/2}^{i,j})\circ \chi_{\Delta t}\circ \prod_{(i,j)\in C^{*}}(\gamma_{\Delta t/2}^{i,j}\circ \phi_{\Delta t/2}^{i,j}),$$

where *C* is an ordering of pairs and *C** is the reverse ordering. While advancing *ξ* by *χ*_Δ*t*_, it is assumed that ***y*** is fixed and, therefore, *h*(***y***) is a constant. This means the dynamics of *ξ*(*t*) is just an Ornstein–Uhlenbeck process with mean *h*/*γ* and unit variance. The exact solution is given by

$$\chi_{\Delta t}\xi_{0}=\xi_{0}e^{-\gamma \Delta t}+\frac{h}{\gamma }(1-e^{-\gamma \Delta t})+e^{-\gamma \Delta t}W(e^{2\gamma \Delta t}-1).$$

The integration of the perturbed dynamics for a vortex pair (*i*,*j*) means integrating the system of ordinary differential equations given by

$$\begin{array}{rl} {\dot{\text{x}}}_{i} & =\frac{\Gamma_{j}}{4\pi }{\text{x}}_{i}\times {\text{x}}_{i}\times {\text{x}}_{j}=\frac{\Gamma_{i}+\Gamma_{j}}{4\pi }{\text{x}}_{i}\times {\text{x}}_{i}\times {\text{x}}_{j}+\frac{\Gamma_{i}-\Gamma_{j}}{4\pi }{\text{x}}_{i}\times {\text{x}}_{i}\times {\text{x}}_{j},\\ {\dot{\text{x}}}_{j} & =\frac{\Gamma_{i}}{4\pi }{\text{x}}_{j}\times {\text{x}}_{j}\times {\text{x}}_{i}=\frac{\Gamma_{i}+\Gamma_{j}}{4\pi }{\text{x}}_{j}\times {\text{x}}_{j}\times {\text{x}}_{i}-\frac{\Gamma_{i}-\Gamma_{j}}{4\pi }{\text{x}}_{j}\times {\text{x}}_{j}\times {\text{x}}_{i}. \end{array}$$

The equations have been split into a symmetric and an antisymmetric part. A symmetric composition may once more be applied to integrate the two parts. In our case, however, the thermostatted system consists only of vortices with strength ±*Γ*_strong_ and thus each vortex pair interaction is either fully symmetric or fully antisymmetric.

By introducing *α*_*S*_=(*Γ*_*i*_+*Γ*_*j*_)/4*π*, the symmetric part may be written as

A 2 $${\dot{\text{x}}}_{i}=\alpha_{S}{\text{x}}_{i}\times {\text{x}}_{i}\times {\text{x}}_{j}=\alpha_{S}({\text{x}}_{j}\times {\text{x}}_{i})\times {\text{x}}_{i}$$

and

A 3 $${\dot{\text{x}}}_{j}=\alpha_{S}{\text{x}}_{j}\times {\text{x}}_{j}\times {\text{x}}_{i}=-\alpha_{S}({\text{x}}_{j}\times {\text{x}}_{i})\times {\text{x}}_{j}.$$

These dynamics are symmetric with respect to the plane equidistant between the two vortices. This is because the dynamics are rotations in opposite direction about the same vector ***x***_*j*_×***x***_*i*_ and this vector must lie in said plane. Furthermore, the dynamics of (7.2)–(7.3) does not allow the vortex pair to pass through the position where they are exactly opposed, as in this case their cross product is zero. All in all, this means the final position of the two vortices is uniquely determined by their chord distance.

The change in the distance between the two vortices can be represented by the change of their inner product

$$\begin{array}{rl} \frac{\partial }{\partial t}({\text{x}}_{i}\cdot {\text{x}}_{j}) & ={\dot{\text{x}}}_{i}\cdot {\text{x}}_{j}+{\text{x}}_{i}\cdot {\dot{\text{x}}}_{j}\\ & =2\alpha_{S}({\left({\text{x}}_{i}\cdot {\text{x}}_{j}\right)}^{2}-1). \end{array}$$

The solution to this differential equation is given by

$${\text{x}}_{i}\cdot {\text{x}}_{j}{|}_{t=\Delta t}=\tanh(2\alpha \Delta t-\mathrm{artanh}({\text{x}}_{i}\cdot {\text{x}}_{j}){|}_{t=0}).$$

In the antisymmetric part, we introduce *α*_*A*_=(*Γ*_*i*_−*Γ*_*j*_)/4*π* to write

$$\begin{array}{rl} {\dot{\text{x}}}_{i} & =\alpha_{A}{\text{x}}_{i}\times {\text{x}}_{i}\times {\text{x}}_{j}\\ \;\mathrm{and}\;{\dot{\text{x}}}_{j} & =-\alpha_{A}{\text{x}}_{j}\times {\text{x}}_{j}\times {\text{x}}_{i}. \end{array}$$

By rearranging the order of the cross product, we achieve

A 4 $${\dot{\text{x}}}_{i}=\alpha_{A}({\text{x}}_{j}\times {\text{x}}_{i})\times {\text{x}}_{i}={\hat{a}}_{A}{\text{x}}_{i}$$

and

A 5 $${\dot{\text{x}}}_{j}=\alpha_{A}({\text{x}}_{j}\times {\text{x}}_{i})\times {\text{x}}_{j}={\hat{a}}_{A}{\text{x}}_{j}.$$

The vector ***a***_*A*_ implicity defined by (7.4) and (7.5) can simply be shown to be constant under the antisymmetric flow. This means the dynamics of the antisymmetric part may be integrated by using Rodigues’s formula.

## References

[1] Schlick T 2010 *Molecular modeling and simulation: an interdisciplinary guide (interdisciplinary applied mathematics)*, 2nd edn Berlin, Germany: Springer.

[2] Frenkel D, Smit B 2002 *Understanding molecular simulation*, 2nd edn San Diego, CA: Academic Press.

[3] Ilg P, Karlin IV, Öttinger HC 2002 Canonical distribution functions in polymer dynamics. (I). Dilute solutions of flexible polymers. *Phys. A* 315, 367–385. (doi:10.1016/S0378-4371(02)01017-8)

[4] Holmes P, Lumley JL, Berkooz G 1998 *Turbulence, coherent structures, dynamical systems and symmetry*. Cambridge, UK: Cambridge University Press.

[5] Davidson PA 2004 *Turbulence: an introduction for scientists and engineers*. Oxford, UK: Oxford University Press.

[6] Hayes W, Jackson KR 2007 A fast shadowing algorithm for high dimensional ODE systems. *SIAM J. Sci. Comput.* 29, 1738–1758. (doi:10.1137/060654840)

[7] Leimkuhler B, Reich S 2005 *Simulating Hamiltonian dynamics*, 1st edn Cambridge, UK: Cambridge University Press.

[8] Hairer E, Lubich C, Wanner G 2006 *Geometric numerical integration: structure-preserving algorithms for ordinary differential equations*. Berlin, Germany: Springer.

[9] Abramov RV, Majda AJ 2003 Statistically relevant conserved quantities for truncated quasigeostrophic flow. *Proc. Natl Acad. Sci. USA* 100, 3841–3846. (doi:10.1073/pnas.0230451100) 12642678 10.1073/pnas.0230451100 PMC404468

[10] Dubinkina S, Frank J 2007 Statistical mechanics of Arakawa’s discretizations. *J. Comput. Phys.* 227, 1286–1305. (doi:10.1016/j.jcp.2007.09.002)

[11] Dubinkina S, Frank J 2010 Statistical relevance of vorticity conservation in the Hamiltonian particle-mesh method. *J. Comput. Phys.* 229, 2634–2648. (doi:10.1016/j.jcp.2009.12.012)

[12] Metropolis N, Rosenbluth AW, Rosenbluth MN, Teller AH, Teller E 1953 Equation of state calculations by fast computing machines. *J. Chem. Phys.* 21, 1087–1092. (doi:10.1063/1.1699114)

[13] Roberts GO, Tweedie RL 1996 Exponential convergence of Langevin distributions and their discrete approximations. *Bernoulli* 2, 341–363. (doi:10.2307/3318418)

[14] Nosé S 1984 A molecular dynamics method for simulations in the canonical ensemble. *Mol. Phys.* 52, 255–268. (doi:10.1080/00268978400101201)

[15] Nosé S 1984 A unified formulation of the constant temperature molecular dynamics methods. *J. Chem. Phys.* 81, 511–519. (doi:10.1063/1.447334)

[16] Dubinkina S, Frank J, Leimkuhler B 2010 Simplified modelling of a thermal bath system, with application to a fluid vortex system. *Multiscale Model Simul.* 8, 1882–1901. (doi:10.1137/100795152)

[17] Ding N, Fang Y, Babbush R, Chen C, Skeel RD, Neven H 2014 Bayesian sampling using stochastic gradient thermostats. In *Advances in neural information processing systems (NIPS)*. Cambridge, MA: MIT Press.

[18] Jaynes ET 1957 Information theory and statistical mechanics. *Phys. Rev.* 106, 620 (doi:10.1103/PhysRev.106.620)

[19] Jaynes ET 1957 Information theory and statistical mechanics. II. *Phys. Rev.* 108, 171–190. (doi:10.1103/PhysRev.108.171)

[20] Panagiotis S 2005 A maximum likelihood algorithm for the estimation and renormalization of exponential densities. *J. Comput. Phys.* 208, 691–703. (doi:10.1016/j.jcp.2005.03.001)

[21] Giffin A, Urniezius R 2014 Simultaneous state and parameter estimation using maximum relative entropy with nonhomogenous differential equation constraints. *Entropy* 16, 4974–4991. (doi:10.3390/e16094974)

[22] Majda AJ, Gershgorin B 2011 Improving model fidelity and sensitivity for complex systems through empirical information theory. *Proc. Natl Acad. Sci. USA* 108, 10 044–10 049. (doi:10.1073/pnas.1105174108) 10.1073/pnas.1105174108 PMC3121845 21646534

[23] Shell MS 2008 The relative entropy is fundamental to multiscale and inverse thermodynamic problems. *J. Chem. Phys.* 129, 144108 (doi:10.1063/1.2992060) 19045135 10.1063/1.2992060

[24] Dama JF, Sinitskiy AV, McCullagh M, Weare J, Roux B, Dinner AR, Voth GA 2013 The theory of ultra-coarse-graining. 1. General principles. *J. Chem. Theory Comput.* 9, 2466–2480. (doi:10.1021/ct4000444) 26583735 10.1021/ct4000444

[25] Agmon N, Alhassid Y, Levine RD 1979 An algorithm for finding the distribution of maximal entropy. *J. Comput. Phys.* 30, 250–258. (doi:10.1016/0021-9991(79)90102-5)

[26] Haken H 2006 *Information and self-organization: a macroscopic approach to complex systems*. Berlin, Germany: Springer.

[27] Davis S, Gutiérrez G 2012 Conjugate variables in continuous maximum-entropy inference. *Phys. Rev. E* 86, 051136 (doi:10.1103/PhysRevE.86.051136) 10.1103/PhysRevE.86.051136 23214767

[28] Samaey G, Lelievre T, Legat V 2011 A numerical closure approach for kinetic models of polymeric fluids: exploring closure relations for fene dumbbells. *Comput. Fluids* 43, 119–133. (doi:10.1016/j.compfluid.2010.06.023)

[29] Mattingly JC, Stuart AM, Higham DJ 2002 Ergodicity for SDEs and approximations: locally Lipschitz vector fields and degenerate noise. *Stoch. Process. Appl.* 101, 185–232. (doi:10.1016/S0304-4149(02)00150-3)

[30] DelSole T 2000 A fundamental limitation of Markov models. *J. Atmos. Sci.* 57, 2158–2168. (doi:10.1175/1520-0469(2000)057<2158:AFLOMM>2.0.CO;2)

[31] Leimkuhler B, Noorizadeh E, Penrose O 2011 Comparing the efficiencies of stochastic isothermal molecular dynamics models. *J. Stat. Phys.* 143, 921–942. (doi:10.1007/s10955-011-0210-2)

[32] Hoover WG 1985 Canonical dynamics: equilibrium phase-space distributions. *Phys. Rev. A* 31, 1695 (doi:10.1103/PhysRevA.31.1695) 10.1103/physreva.31.1695 9895674

[33] Samoletov A, Dettmann C, Chaplain M 2007 Thermostats for ‘slow’ configurational modes. *J. Stat. Phys.* 128, 1321–1336. (doi:10.1007/s10955-007-9365-2)

[34] Bulgac A, Kusnezov D 1990 Canonical ensemble averages from pseudomicrocanonical dynamics. *Phys. Rev. A* 42, 5045–5048. (doi:10.1103/PhysRevA.42.5045) 9904625 10.1103/physreva.42.5045

[35] Leimkuhler B 2010 Generalized Bulgac-Kusnezov methods for sampling of the Gibbs-Boltzmann measure. *Phys. Rev. E* 81, 026703 (doi:10.1103/PhysRevE.81.026703) 10.1103/PhysRevE.81.026703 20365671

[36] Bajars J, Frank JE, Leimkuhler B 2013 Weakly coupled heat bath models for Gibbs-like invariant states in nonlinear wave equations. *Nonlinearity* 26, 1945 (doi:10.1088/0951-7715/26/7/1945)

[37] Bajars J, Frank J, Leimkuhler B 2011 Stochastic-dynamical thermostats for constraints and stiff restraints. *Eur. Phys. J. Spec. Topics* 200, 131–152. (doi:10.1140/epjst/e2011-01522-0)

[38] Dewar RC, Lineweaver CH, Niven RK, Regenauer-Lieb K (eds) 2014 Beyond the second law: entropy production and non-equilibrium systems. In *Understanding complex systems*. Berlin, Germany: Springer.

[39] Majda AJ, Wang X 2006 *Nonlinear dynamics and statistical theories for basic geophysical flows*. Cambridge, UK: Cambridge University Press.

[40] Balian R 2006 *From microphysics to macrophysics: methods and applications of statistical physics*, vol. 2 Berlin, Germany: Springer.

[41] Cottet G-H, Koumoutsakos PD 2000 *Vortex methods*, 1st edn Cambridge, UK: Cambridge University Press.

[42] Newton PK 2001 *The N-vortex problem: analytical techniques*, 1st edn New York, NY: Springer.

[43] Majda AJ, Bertozzi AL 2002 *Vorticity and incompressible flow*, 1st edn Cambridge, UK: Cambridge University Press.

[44] Newton PK, Shokraneh H 2006 The N-vortex problem on a rotating sphere. I. Multi-frequency configurations. *Proc. R. Soc. A* 462, 149–169. (doi:10.1098/rspa.2005.1566)

[45] Jamaloodeen MI, Newton PK 2006 The N-vortex problem on a rotating sphere. II. Heterogeneous platonic solid equilibria. *Proc. R. Soc. A* 462, 3277–3299. (doi:10.1098/rspa.2006.1731)

[46] Newton PK, Sakajo T 2007 The N-vortex problem on a rotating sphere. III. Ring configurations coupled to a background field. *Proc. R. Soc. A* 463, 961–977. (doi:10.1098/rspa.2006.1802)

[47] Laurent-Polz F 2005 Point vortices on a rotating sphere. *Regul. Chaotic Dyn.* 1, 39–58. (doi:10.1070/RD2005v010n01ABEH000299)

[48] Myerscough KW, Frank J 2016 Explicit, parallel Poisson integration of point vortices on the sphere. *J. Comput. Appl. Math.* 304, 100–119. (doi:10.1016/j.cam.2016.02.053)

[49] Faddeev LD, Takhtajan LA 1987 *Hamiltonian methods in the theory of solitons*. Berlin, Germany: Springer.

[50] Frank J 2004 Geometric space–time integration of ferromagnetic materials. *Appl. Numer. Math.* 48, 307–322. (doi:10.1016/j.apnum.2003.11.003)

[51] Frank J, Huang W, Leimkuhler B 1997 Geometric integrators for classical spin systems. *J. Comput. Phys.* 133, 160–172. (doi:10.1006/jcph.1997.5672)

[52] Kehrbaum S, Maddocks JH 1997 Elasitc rods, rigid bodies, quaternions and the last quadrature. *Phil. Trans. R. Soc. Lond. A* 355, 2117–2136. (doi:10.1098/rsta.1997.0113)

[53] Zhang M-Q, Qin M-Z 1993 Explicit symplectic schemes to solve vortex systems. *Comput. Math. Appl.* 26, 51–56. (doi:10.1016/0898-1221(93)90073-5)

[54] Patrick GW 2000 Dynamics of perturbed relative equilibria of point vortices on the sphere or plane. *J. Nonlin. Sci.* 10, 401–415. (doi:10.1007/s003329910015)

[55] Bloch A, Krishnaprasad PS, Marsden JE, Ratiu TS 1996 The Euler-Poincaré equations and double bracket dissipation. *Commun. Math. Phys.* 175, 1–42. (doi:10.1007/BF02101622)

[56] Kliemann W 1987 Recurrence and invariant measures for degenerate diffusions. *Ann. Probab.* 15, 690–707. (doi:10.1214/aop/1176992166)

[57] Rey-Bellet L 2006 Ergodic properties of Markov processes. In *Open quantum systems II*, pp. 1–39. Berlin, Heidelberg, Germany: Springer.

[58] Bühler O 2002 Statistical mechanics of strong and weak point vortices in a cylinder. *Phys. Fluids* 14, 2139–2149. (doi:10.1063/1.1483305)

[59] Chavanis P-H 2001 Kinetic theory of point vortices: diffusion coefficient and systematic drift. *Phys. Rev. E* 64, 026309 (doi:10.1103/PhysRevE.64.026309) 10.1103/PhysRevE.64.026309 11497701

[60] Cotter CJ, Pavliotis GA 2009 Estimating eddy diffusivities from noisy Lagrangian observations. *Commun. Math. Sci.* 7, 805–838. (doi:10.4310/CMS.2009.v7.n4.a2)

[61] Pavliotis GA, Stuart AM 2008 *Multiscale methods: averaging and homogenization*. Berlin, Germany: Springer.

[62] Hornung U 1997 *Homogenization and porous media*, vol. 6 Berlin, Germany: Springer.

[63] Frank J, Gottwald GA 2011 The Langevin limit of the Nosé-Hoover-Langevin thermostat. *J. Stat. Phys.* 143, 715–724. (doi:10.1007/s10955-011-0203-1)

