# Supplementary figures and images for: Observation-based correction of dynamical models using thermostats
Source: Proc Math Phys Eng Sci. 2017 Jan;473(2197):20160730. doi: 10.1098/rspa.2016.0730 (PMC5312133; doi:10.1098/rspa.2016.0730)

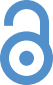

Supplement: Observation-based-correction.zip [file rspa20160730supp8.zip › RSTA_OpenAccesslogo_RGB.pdf]

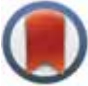

CrossMark

[click for updates](#)

Supplement: Observation-based-correction.zip [file rspa20160730supp8.zip › RS_crossmark_logo.pdf]

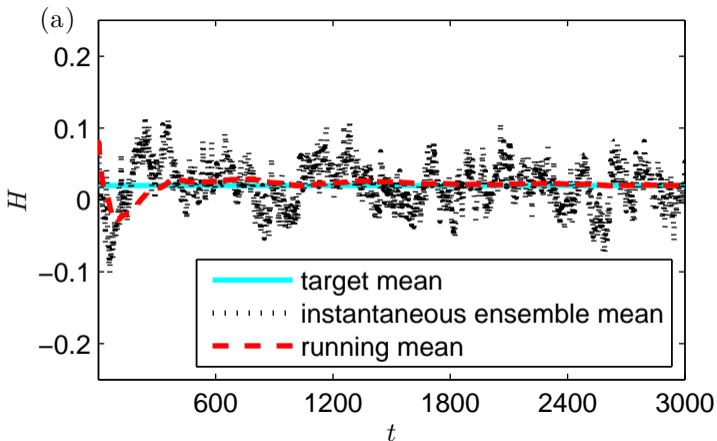

Supplement: Observation-based-correction.zip [file rspa20160730supp8.zip › images/H_neutral_follow1_K2.pdf]

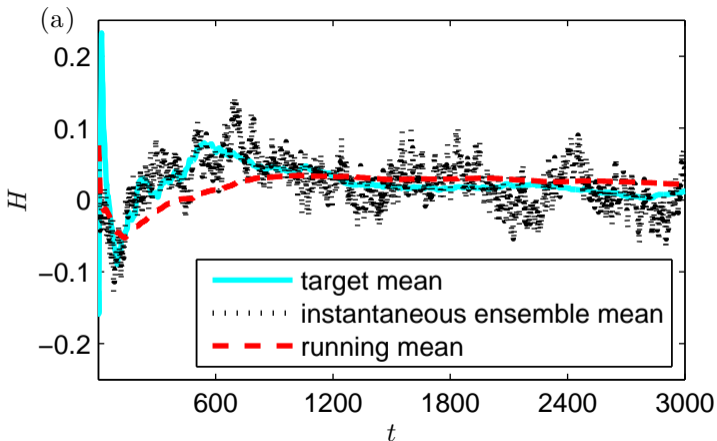

Supplement: Observation-based-correction.zip [file rspa20160730supp8.zip › images/H_neutral_follow2_K2.pdf]

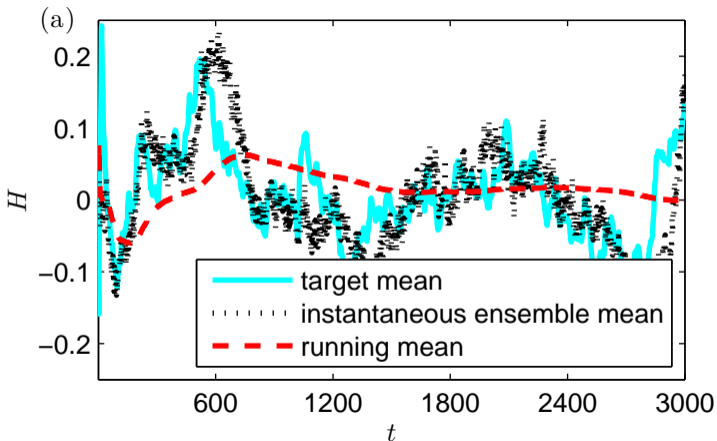

Supplement: Observation-based-correction.zip [file rspa20160730supp8.zip › images/H_neutral_follow4_K2.pdf]

(b)

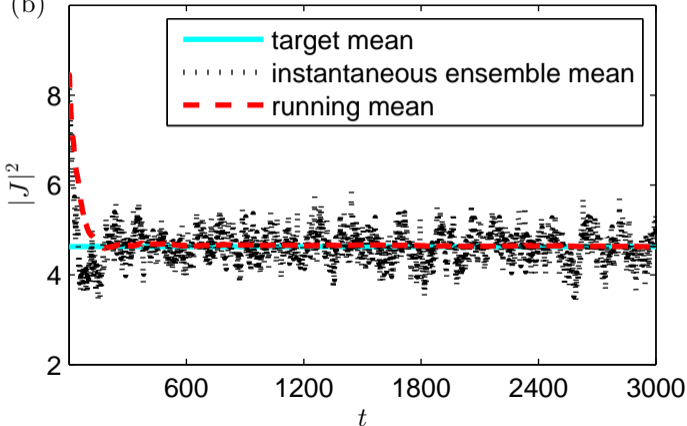

Supplement: Observation-based-correction.zip [file rspa20160730supp8.zip › images/J_neutral_follow1_K2.pdf]

(b)

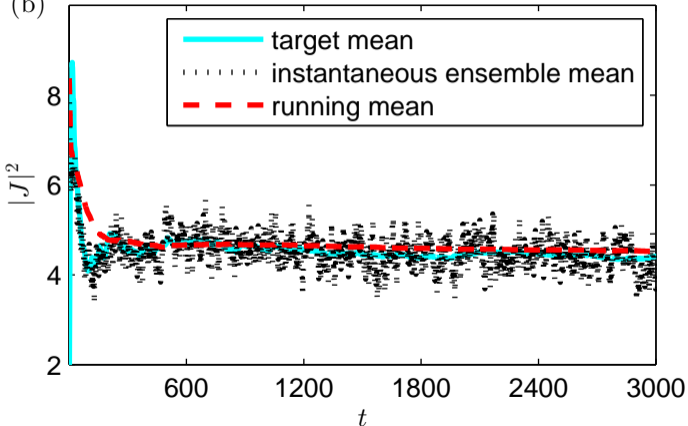

Supplement: Observation-based-correction.zip [file rspa20160730supp8.zip › images/J_neutral_follow2_K2.pdf]

(b)

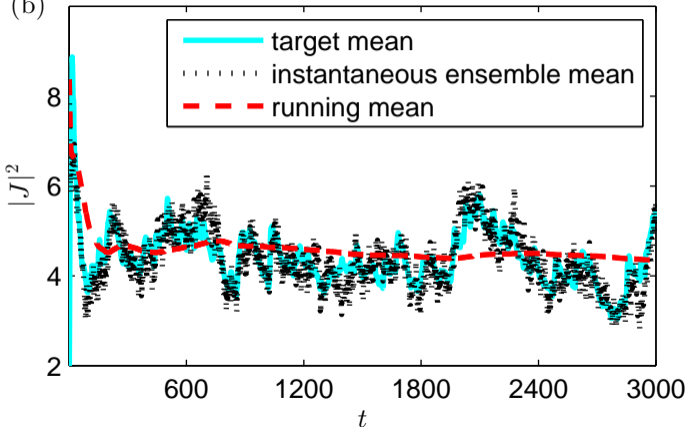

Supplement: Observation-based-correction.zip [file rspa20160730supp8.zip › images/J_neutral_follow4_K2.pdf]

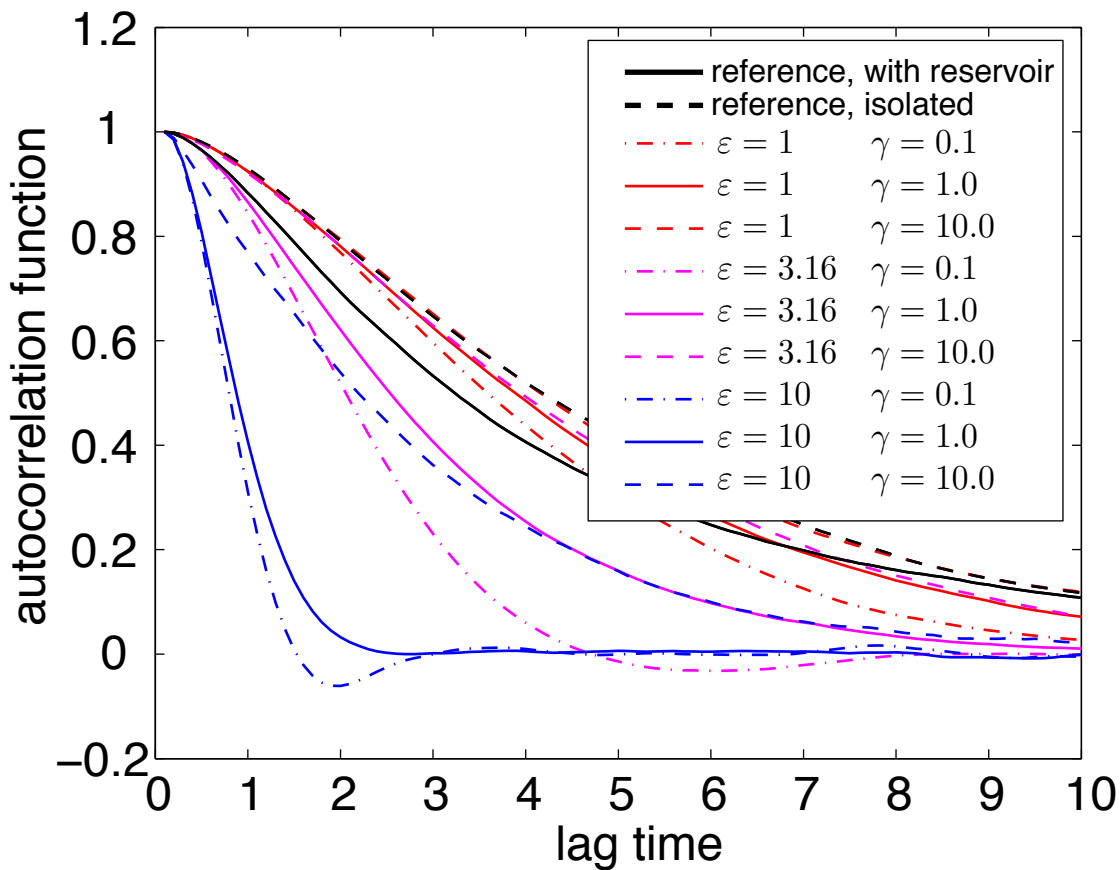

Supplement: Observation-based-correction.zip [file rspa20160730supp8.zip › images/autocorrelation.pdf]

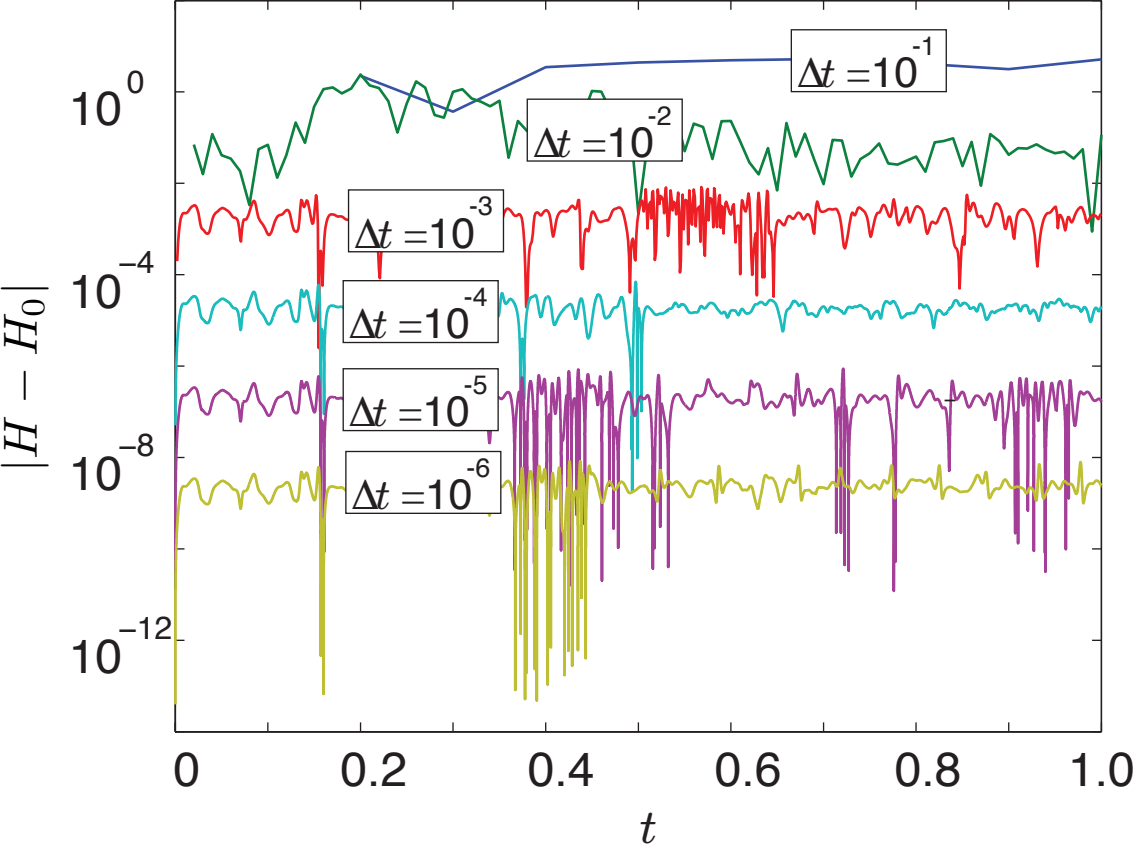

Supplement: Observation-based-correction.zip [file rspa20160730supp8.zip › images/convH_Gadi-2.pdf]

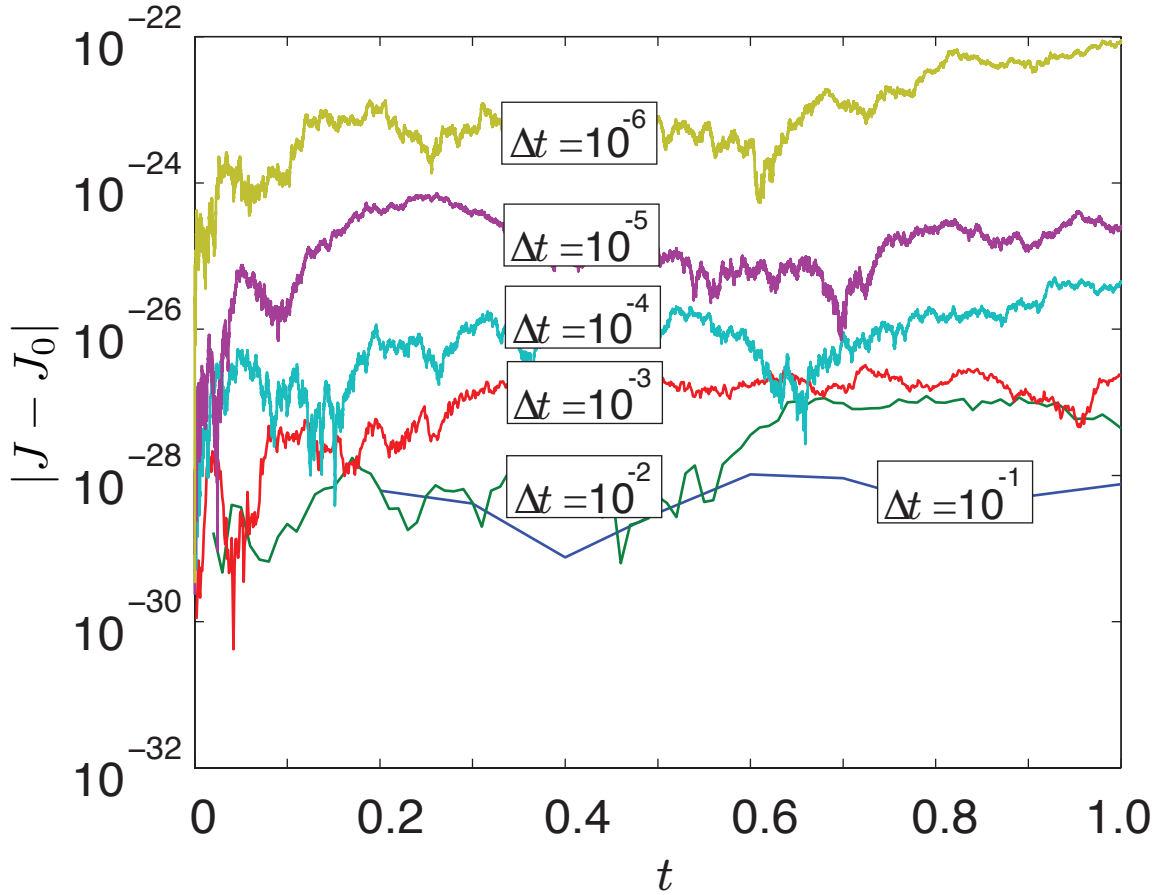

Supplement: Observation-based-correction.zip [file rspa20160730supp8.zip › images/convJ_Gadi-2.pdf]

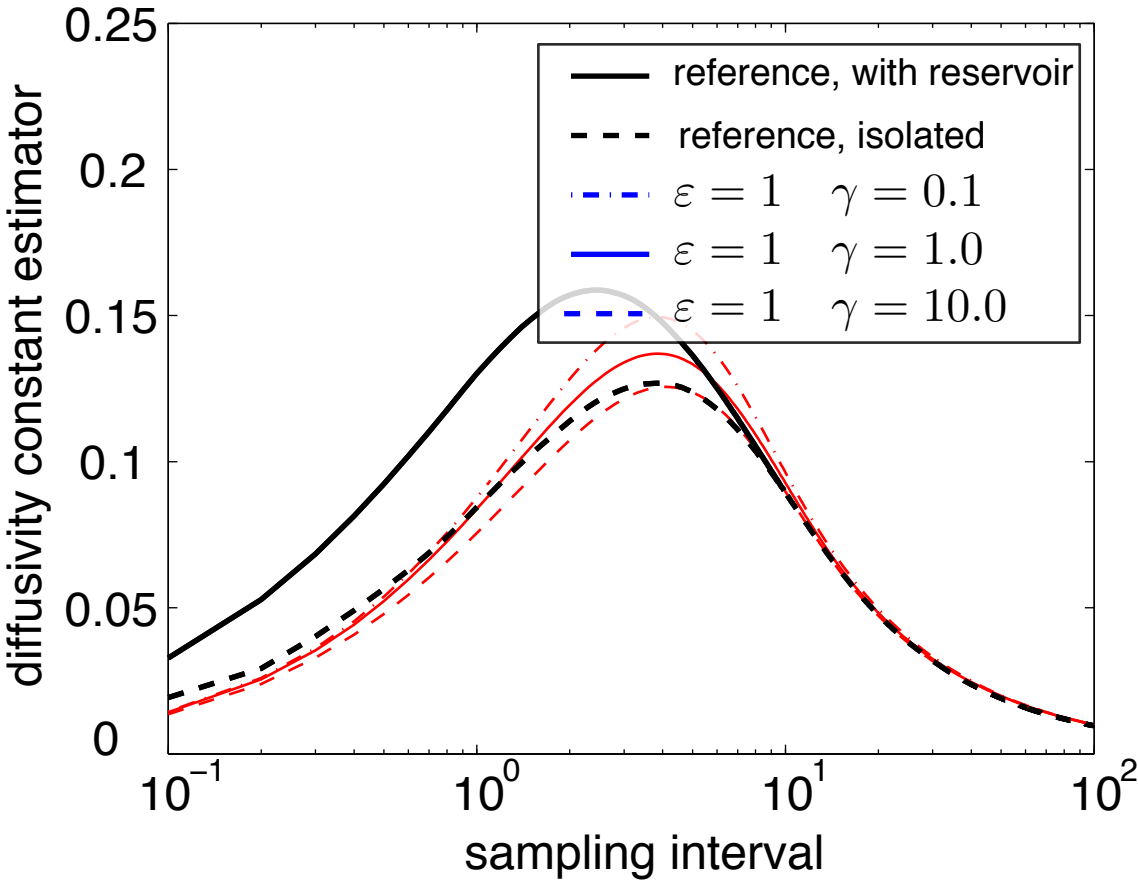

Supplement: Observation-based-correction.zip [file rspa20160730supp8.zip › images/diffusivity_e0-2.pdf]

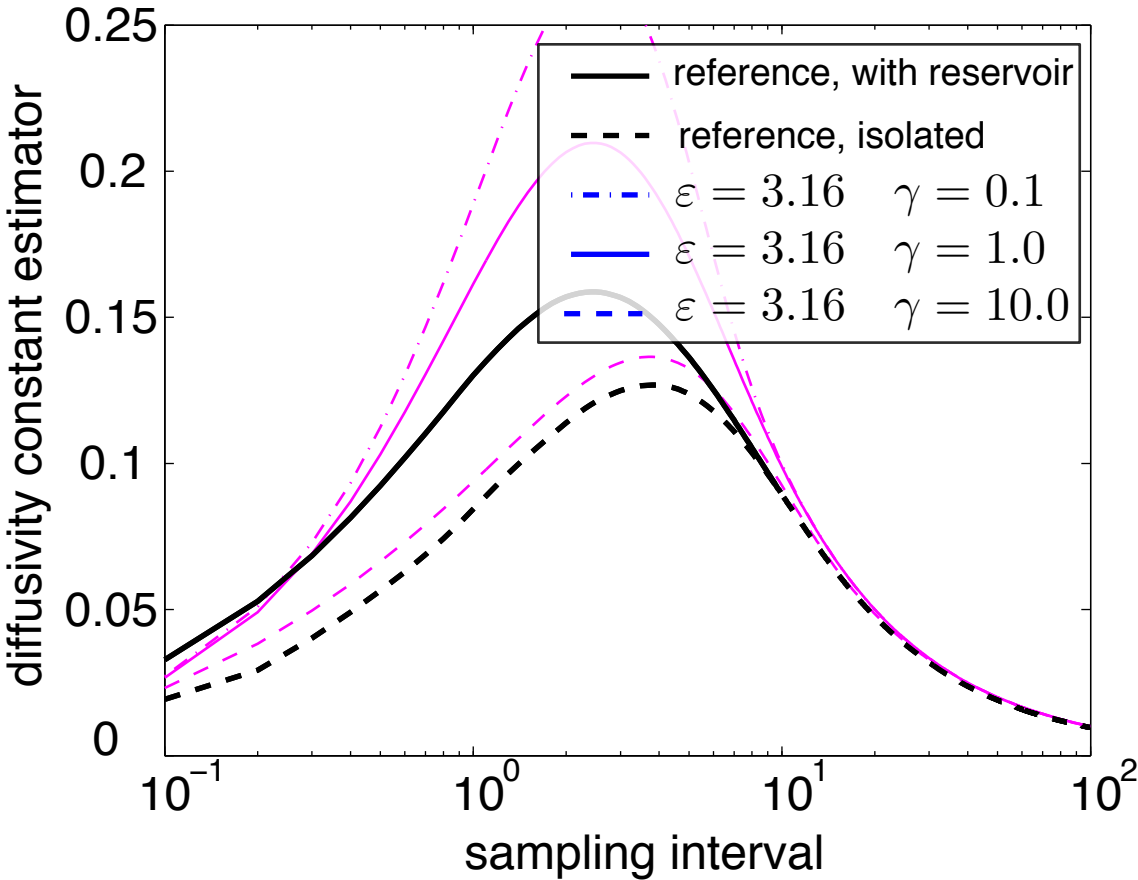

Supplement: Observation-based-correction.zip [file rspa20160730supp8.zip › images/diffusivity_e05-2.pdf]

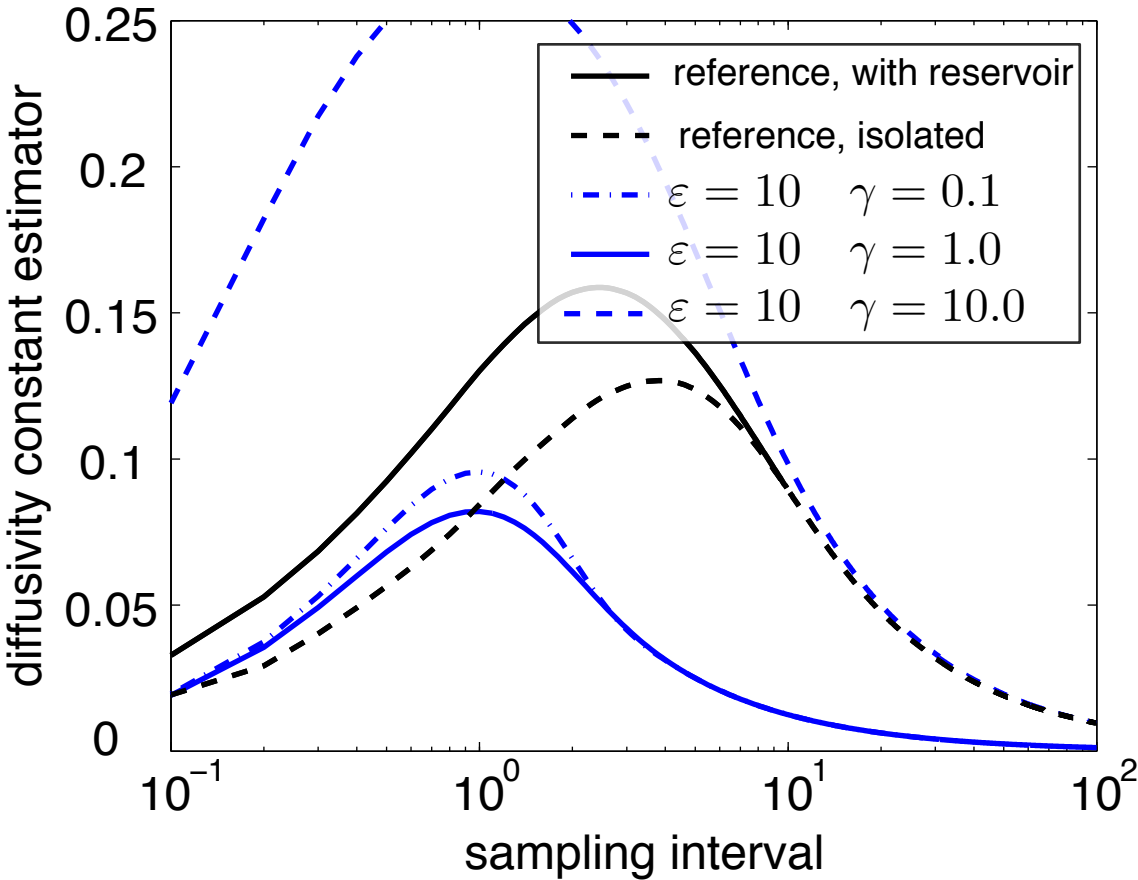

Supplement: Observation-based-correction.zip [file rspa20160730supp8.zip › images/diffusivity_e1-2.pdf]

diffusivity constant estimator

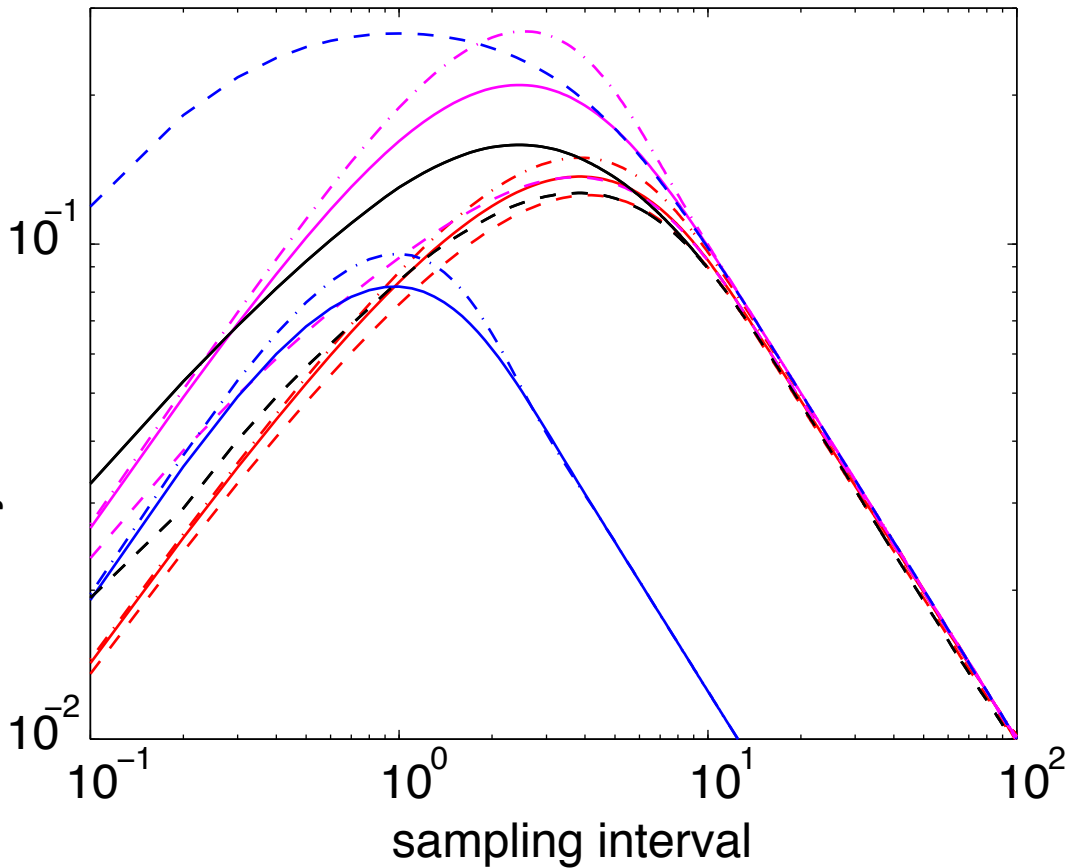

Supplement: Observation-based-correction.zip [file rspa20160730supp8.zip › images/diffusivityloglog-2.pdf]

Energy of the strong vortices

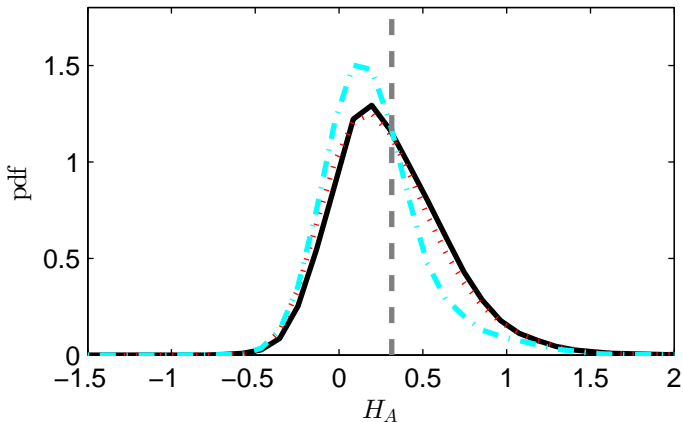

Supplement: Observation-based-correction.zip [file rspa20160730supp8.zip › images/energy_highest.pdf]

Energy of the strong vortices

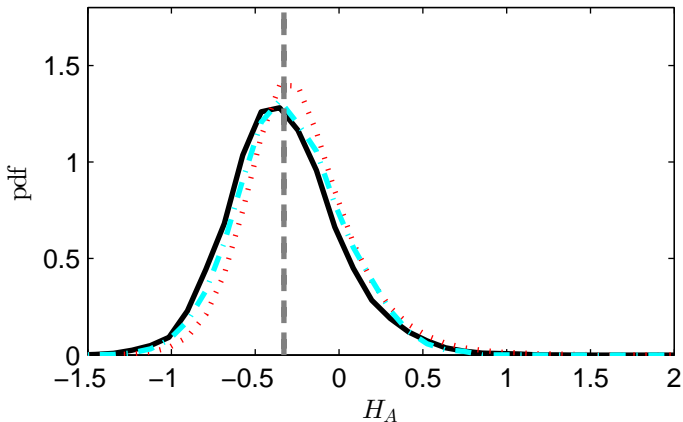

Supplement: Observation-based-correction.zip [file rspa20160730supp8.zip › images/energy_lowest.pdf]

Energy of the strong vortices

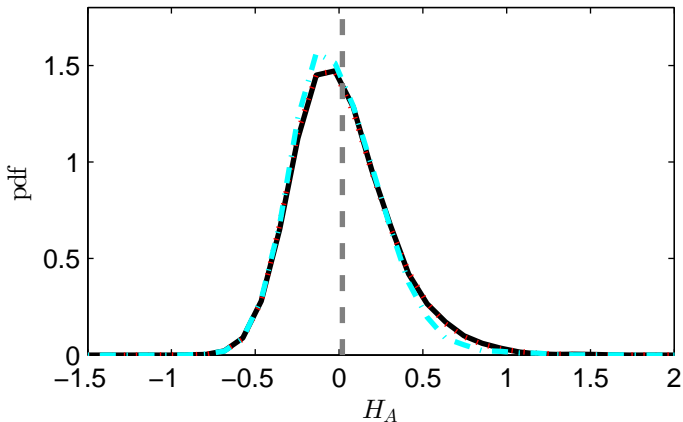

Supplement: Observation-based-correction.zip [file rspa20160730supp8.zip › images/energy_neutral.pdf]

# Momentum magnitude of the strong vortices

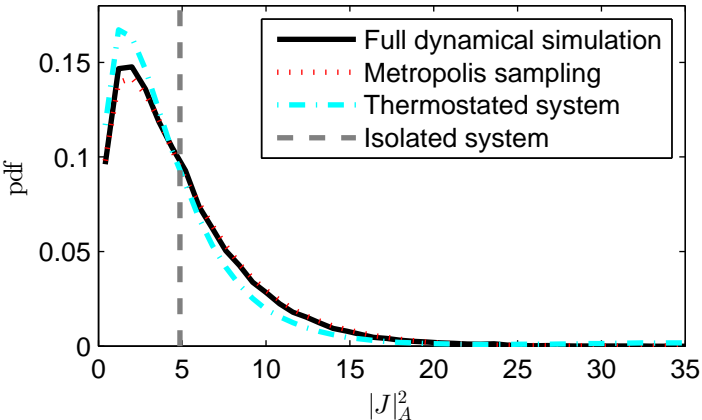

Supplement: Observation-based-correction.zip [file rspa20160730supp8.zip › images/jz_highest.pdf]

Momentum magnitude of the strong vortices

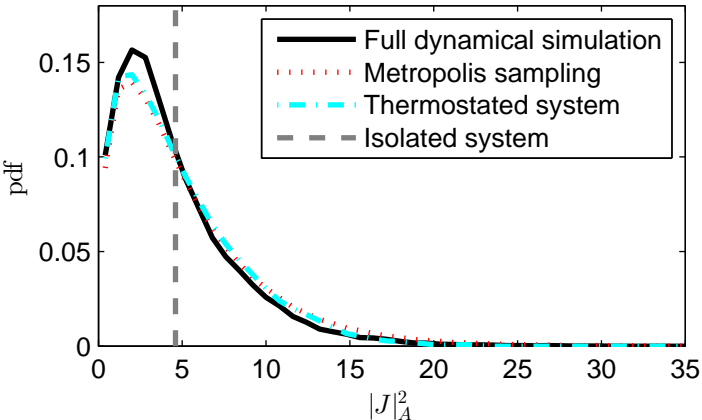

Supplement: Observation-based-correction.zip [file rspa20160730supp8.zip › images/jz_lowest.pdf]

# Momentum magnitude of the strong vortices

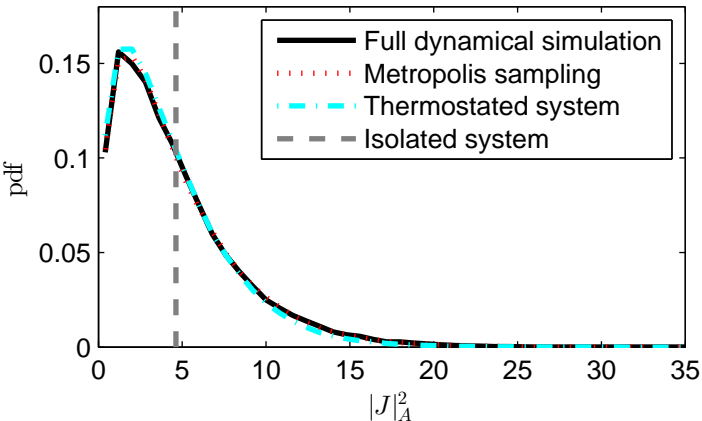

Supplement: Observation-based-correction.zip [file rspa20160730supp8.zip › images/jz_neutral.pdf]

(c)

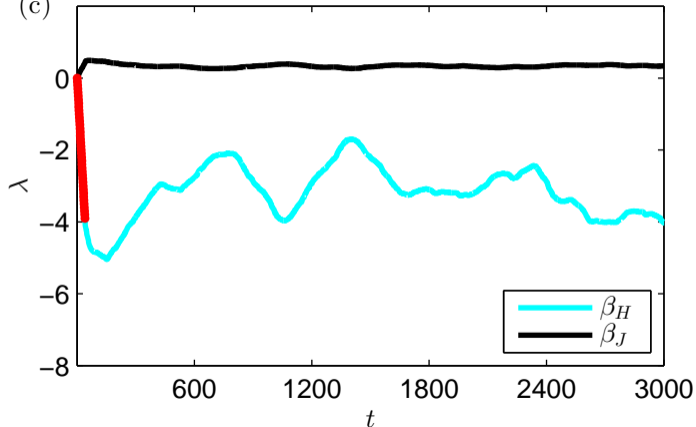

Supplement: Observation-based-correction.zip [file rspa20160730supp8.zip › images/l_withlim_neutral_follow1_K2.pdf]

(c)

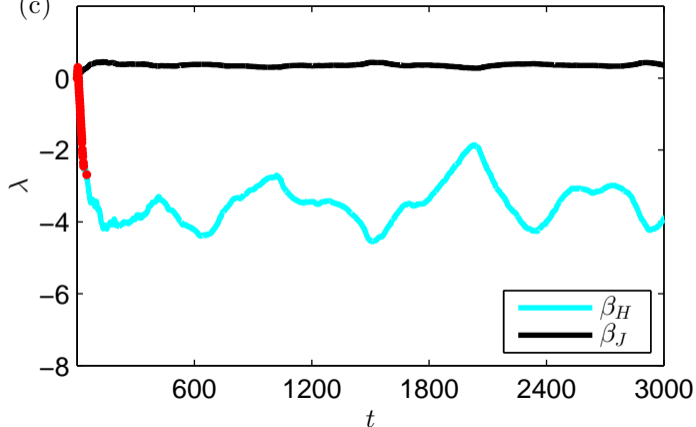

Supplement: Observation-based-correction.zip [file rspa20160730supp8.zip › images/l_withlim_neutral_follow2_K2.pdf]

(c)

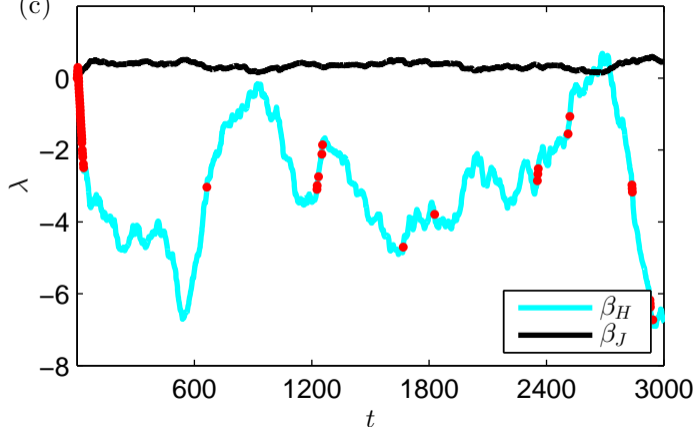

Supplement: Observation-based-correction.zip [file rspa20160730supp8.zip › images/l_withlim_neutral_follow4_K2.pdf]

distance between like signed vortices

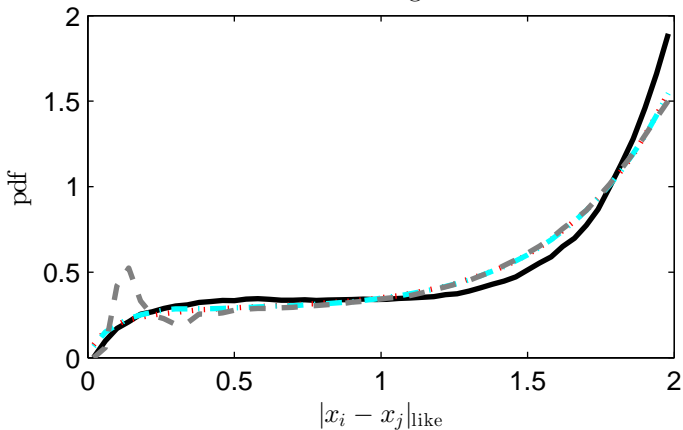

Supplement: Observation-based-correction.zip [file rspa20160730supp8.zip › images/like_highest.pdf]

distance between like signed vortices

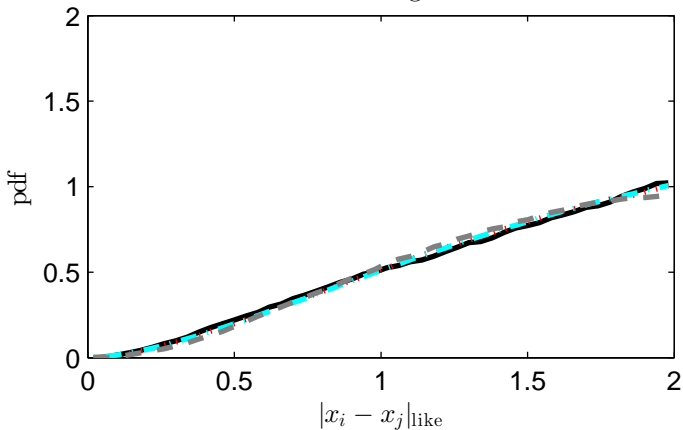

Supplement: Observation-based-correction.zip [file rspa20160730supp8.zip › images/like_lowest.pdf]

distance between like signed vortices

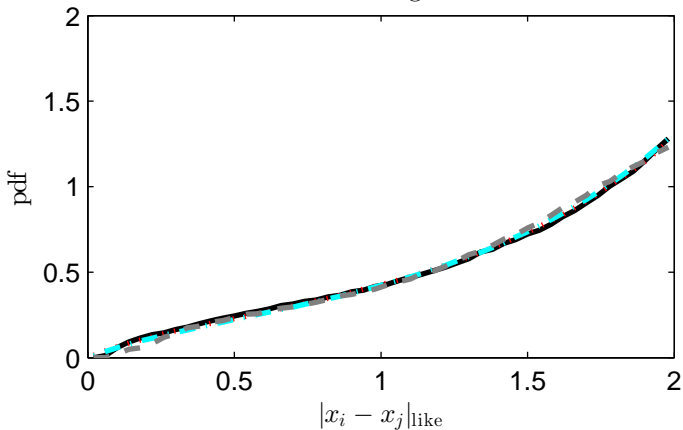

Supplement: Observation-based-correction.zip [file rspa20160730supp8.zip › images/like_neutral.pdf]

distance between opposite signed vortices

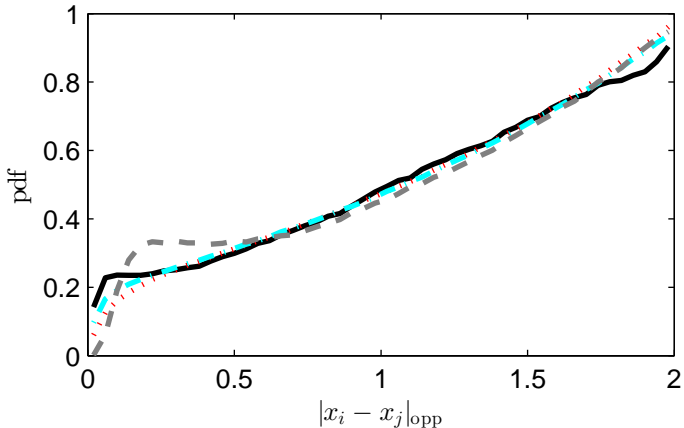

Supplement: Observation-based-correction.zip [file rspa20160730supp8.zip › images/opp_lowest.pdf]

distance between opposite signed vortices

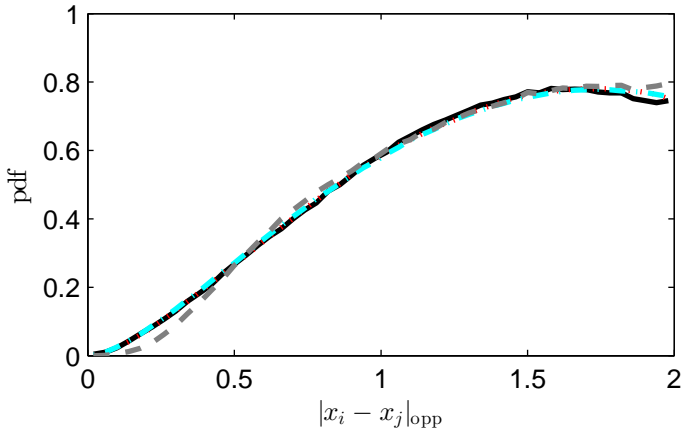

Supplement: Observation-based-correction.zip [file rspa20160730supp8.zip › images/opp_neutral.pdf]
