# Supplementary material for: Observation-based correction of dynamical models using thermostats
Source: Proc Math Phys Eng Sci. 2017 Jan;473(2197):20160730. doi: 10.1098/rspa.2016.0730 (PMC5312133; doi:10.1098/rspa.2016.0730)
Supplement: Observation-based-correction.zip [file opp_highest.pdf › images/opp_highest.pdf]

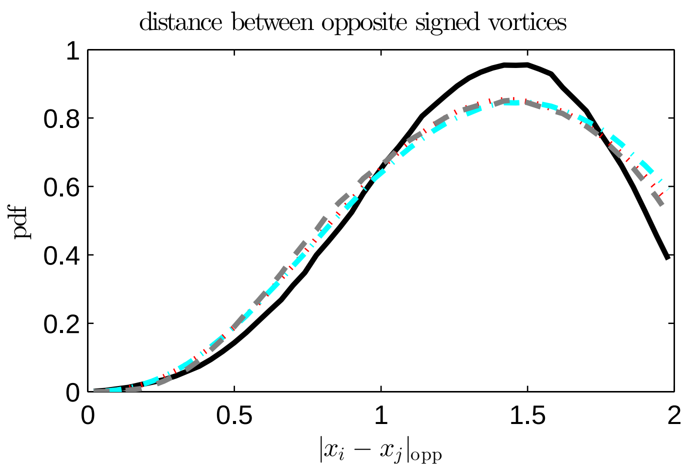
distance between opposite signed vortices
pdf
$|x_i - x_j|_{\mathrm{opp}}$
0
0.2
0.4
0.6
0.8
1
0
0.5
1
1.5
2
